# Changing smoking-mortality association over time and across social groups: National census-mortality cohort studies from 1981 to 2011

**DOI:** 10.1038/s41598-017-11785-x

**Published:** 2017-09-13

**Authors:** Andrea Teng, June Atkinson, George Disney, Nick Wilson, Tony Blakely

**Affiliations:** Department of Public Health, University of Otago, Wellington, New Zealand

## Abstract

The difference in mortality between current and never-smokers varies over time, affecting future projections of health gains from tobacco control. We examine this heterogeneity by sex, ethnicity and cause of death on absolute and relative scales using New Zealand census data. These data included smoking status, and were linked to subsequent mortality records in 1981–84, 1996–99 and 2006–11 for 25–74 year olds (16.1 million person-years of follow-up). Age-standardised mortality rates and rate differences (SRDs) were calculated comparing current to never-smokers, and Poisson regression was used to adjust for multiple socioeconomic factors and household smoking. We found that mortality declined over time in never-smokers; however, mortality trends in current-smokers varied by sex, ethnicity and cause of death. SRDs were stable over time in European/Other men, moderately widened in European/Other women and markedly increased in Māori men and women (Indigenous population). Poisson smoking-mortality rate ratios (RRs) increased from 1981–84 to 1996–99 with a moderate increase from 1996–99 to 2006–11 (RRs 1.48, 1.77, 1.79 in men and 1.51, 1.80, 1.90 in women). Socioeconomic confounding increased over time. In summary, this marked heterogeneity in smoking-mortality RRs over time has implications for estimating the future health and inequality impacts of tobacco control interventions.

## Introduction

**Figure 1:**
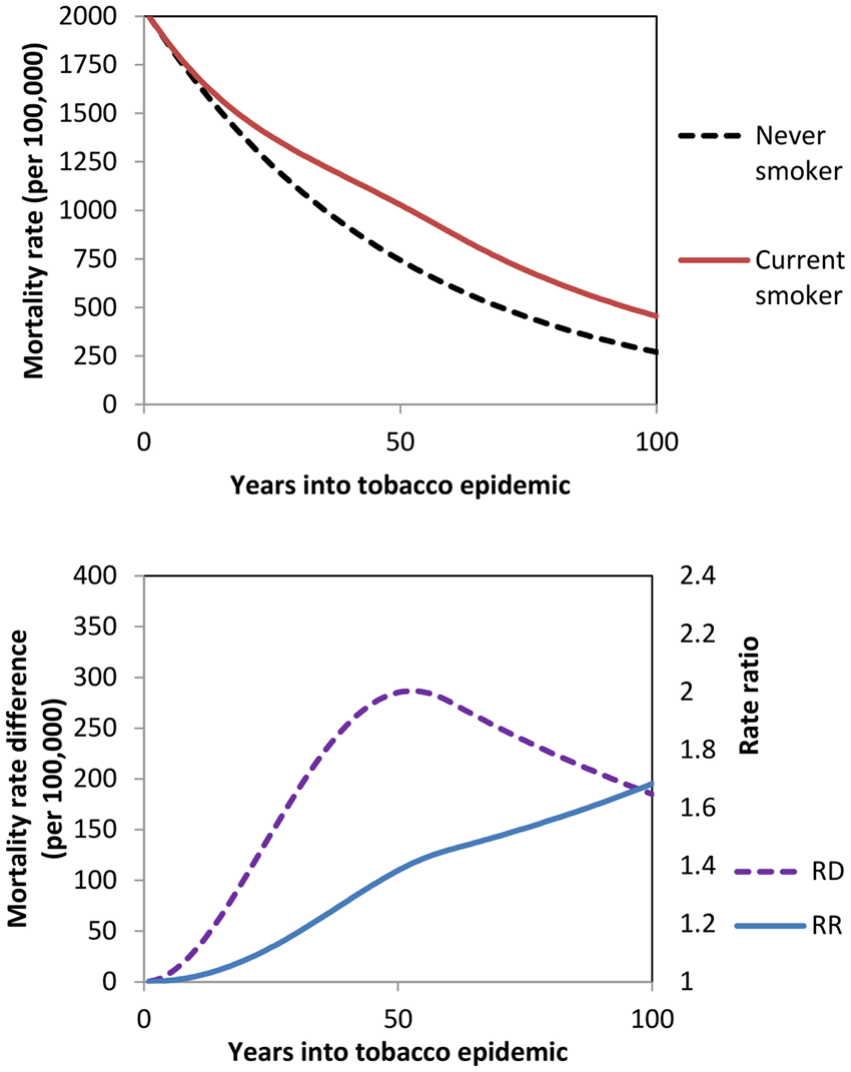
Hypothesised progression of current and never smoker mortality rates (top), and the ﻿corresponding﻿ rate ratios and rate differences (b﻿ottom), over 100 years of the tobacco epidemic.

Tobacco is a causal risk factor for a substantial burden of premature mortality and it is the greatest contributor to ethnic and socioeconomic inequalities in mortality in many high-income countries^1^. However, the mortality burden and social patterning of smoking varies over time^2–6^. It takes decades for the peak harms of smoking to manifest due to time-lags from smoking initiation to disease-specific mortality. Once this time-lag has elapsed, the harms of smoking compared to never-smoking may reduce over time in absolute terms, due to changes in smoking behaviours (eg, number of cigarettes per day) or product toxicity. Conversely, this tobacco-harm often occurs at the same time as long-term downward trends in never-smoker mortality due to other reasons (eg, falling coronary heart disease rates). This dynamic situation means that at least one of the absolute (rate difference, RD) and relative mortality comparisons (rate ratio, RR) between current and never-smokers, will change over time as the tobacco epidemic unfolds. Figure 1 is one possible realisation of this dynamic (others are shown in Supplementary File Appendix A). The relative differences in smoking by mortality (RRs) are likely to go up both early in the tobacco epidemic (due to time-lags from smoking initiation to mortality impact) and also may increase later in the tobacco epidemic (if the fall in never smoker mortality is faster than any fall in the absolute harms of tobacco).

Does this variation in the effect size from smoking, be it either or both on the absolute and relative scales, matter for tobacco control or eradication policy? Almost certainly yes. If one is making a case for tobacco reduction or eradication based on likely future health gains, then having some idea – even quantitative projections – of the future RD and RRs comparing current (and ex) to never smokers is necessary for accurate prediction of mortality reductions and (healthy) life expectancy extensions. Such information might allow policy-makers to more appropriately prioritise tobacco control interventions relative to competing ways to reduce health loss and health inequalities (eg, via obesity prevention interventions).

This theoretical expectation of at least one of the relative and absolute smoking-mortality associations changing over time is supported by recent longitudinal studies of the tobacco epidemic that report increasing relative rates of mortality in current-smokers compared to never-smokers over several decades^7–10^. For example, there were large increases in smoking RRs and RDs for all-cause mortality and lung cancer mortality in the United States (US) between the 1960s, 1980s and 2000s^8^. An artefactual reason for why the smoking-mortality association may vary is increased confounding over time as smoking becomes more strongly patterned by socioeconomic position (SEP), passive smoking^11^ and other behaviours (eg, diet) that are independently associated with mortality. Previous studies are limited in their adjustment for confounding by SEP^8, 10, 12^ (eg, stratifying on education only) and may be susceptible to increased residual confounding over time^13^. This study thoroughly adjusts for confounding by SEP and household smoking (HHS) and the large sample size enables us to examine fine-grained heterogeneity in the data by sex, ethnicity and cause of death over time. Most existing studies are limited to one cohort (with the exception of Thun *et al*.)^8^ and a subset of the population^9, 10, 12^.

The change over time in excess smoking mortality is expected to be differentially phased over time by social groups such as sex, SEP and ethnicity/race. For example, tobacco is classically taken up in a population by men first, then by women^14^. Historically the relative mortality rate in current compared to never-smokers was greater in men than in women but several studies show a convergence in recent cohorts^8, 12^ consistent with epidemic phasing. There are also examples where smoking-mortality rates, RDs and RRs for lung cancer are increasing among women but appear to be reaching a plateau in men^8^. Studies in the United States and New Zealand have also shown notable differences in the smoking-mortality RRs by ethnicity^7, 15^, but little difference on the absolute scale (RD) by ethnicity^7^. Thus while tobacco control appears to be one of the most efficient ways to reduce health inequalities^16^, this too will change over time with the phasing of the tobacco epidemic.

Given this background and rationale, the objectives of this study were to: 1) describe the variation over three decades in the smoking-mortality association on both the absolute and relative scale, by sex and ethnic group; 2) determine the changing contributions of specific causes of death to excess smoking mortality over time; and 3) quantify the increasing confounding by SEP and HHS (the latter as a proxy for passive smoking exposure)^11, 17^ over time. European/Other and Māori populations in New Zealand were selected as a case study, extending a previous study^8^ to examine mortality records from three national census-linked cohorts, with 16.1 million person-years of follow-up, high quality data on smoking, ethnicity and SEP and relatively thorough control of confounding including from household smoking. Māori are the Indigenous peoples of New Zealand and have higher rates of non-communicable disease for multiple reasons – not just tobacco.

## Results

### Variation in SRDs and SRRs over time by population group

**Figure 2:**
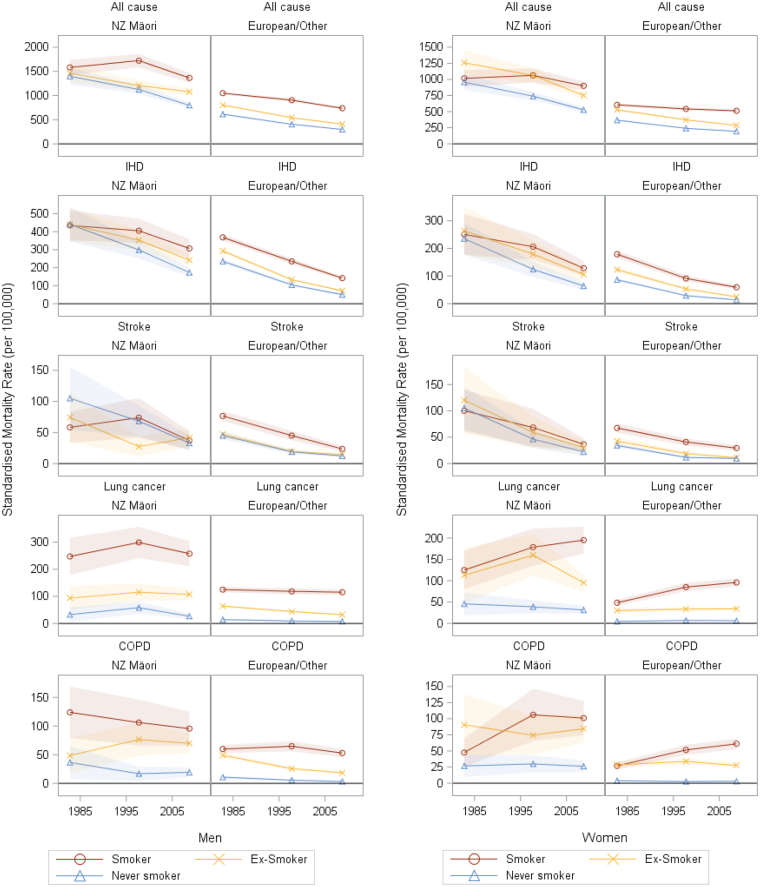
Age-standardised mortality rates by smoking status and ethnicity for men and women aged 25–74 years in the New Zealand Census Mortality Study in 1981–84, 1996–99 and 2006–11 (95% confidence intervals are indicated by shaded bands) for all cause mortality, ischaemic heart disease (IHD, stroke, lung cancer and chronic obstructive pulmonary dis﻿ease (COPD).

**Figure 3:**
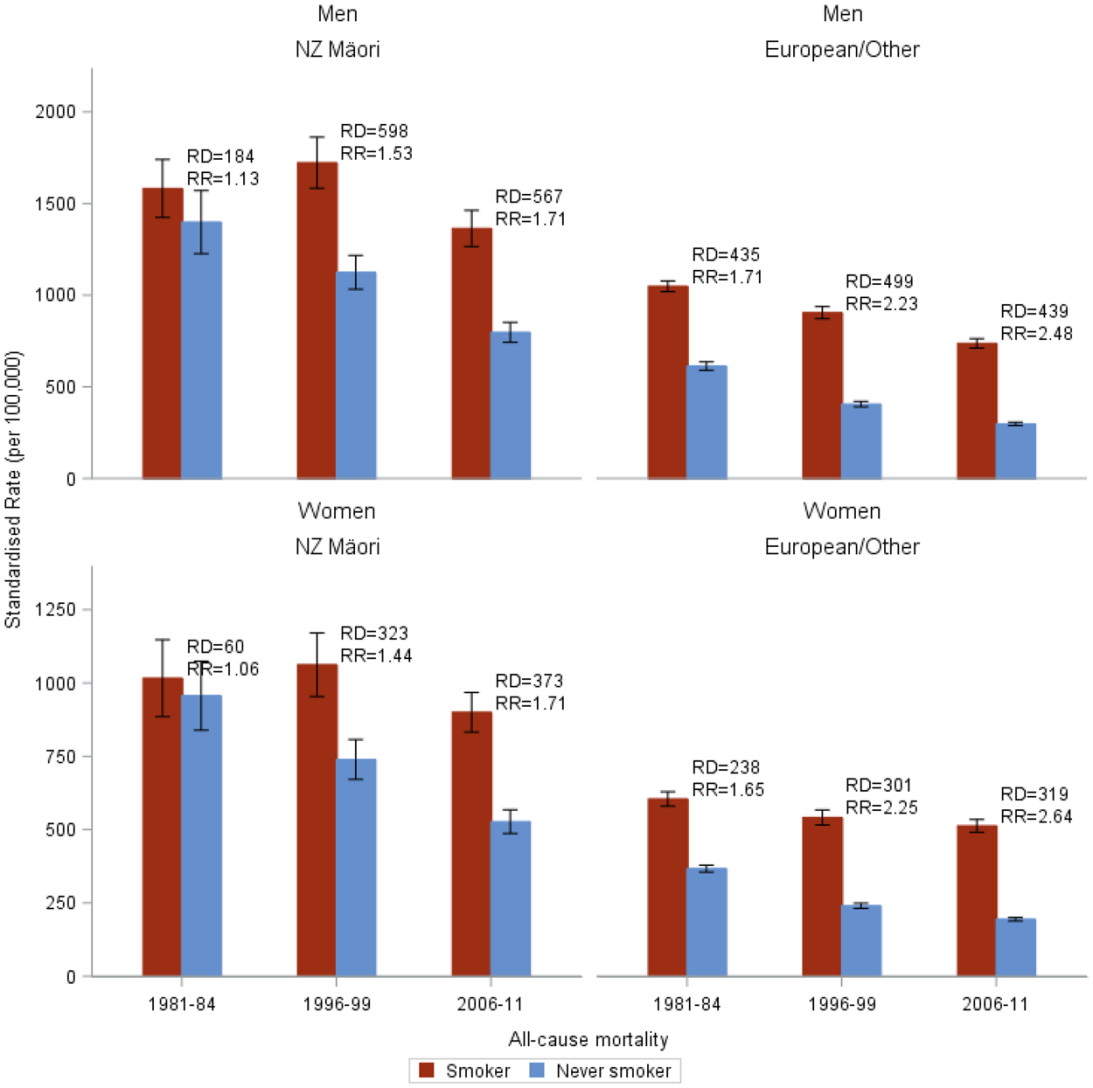
Age-standardised all-cause mortality rates per 100,000 in current-smokers and never-smokers showing the standardised rate differences (RD) and the standardised rate ratios (RR) by cohort and sex in the New Zealand Census Mortality Study (for those aged 25–74 years old).

Figure 2 shows the age-standardised all-cause mortality rates in never- and current-smokers over time. Among European/Other men, current and never-smoker mortality rates have fallen roughly in parallel with little change in SRDs over time (435, 499 and 439 per 100,000), with a corresponding increase in SRRs over time from 1.71 to 2.48 (108% increase in excess SRR (ie, SRR minus 1); Fig. 3). Among European/Other women, the SRDs were less than in men, but widened over time from 238 to 319 per 100,000 (34% increase) with more pronounced widening in SRRs from 1.65 to 2.64 (152% increase in excess RR).

For Māori, there was only a 13% (men) and 6% (women) higher mortality among current versus never-smokers in the early 1980s, but there was an increase in mortality in smokers from the 1980s to 1990s (in comparison to a decrease for never-smokers). This corresponded to a marked increase in both the SRDs and SRRs among Māori men and women in this time period. From 1996–99 to 2006–11, the SRDs were stable (598 and 567 per 100,000 for Māori men, and 323 and 373 for Māori women) and somewhat higher than among European/Other within each sex by time pair. By 2006–11 the SRR was 1.71 among both Māori men and women which was considerably less than among European/Other (2.48 and 2.64 respectively).

### Contributions of specific tobacco-related causes of death

**Figure 4:**
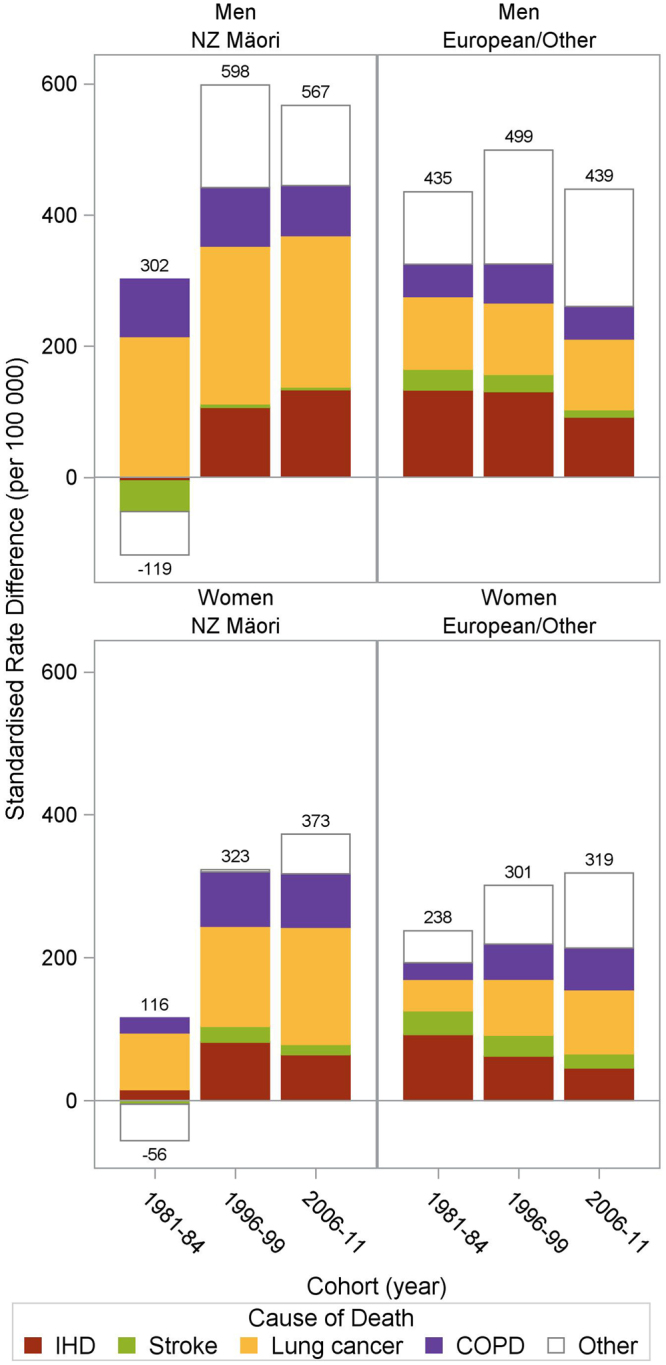
Decomposition of the age-standardised rate differences in mortality between current smokers and never smokers by mortality type in 25–74 year olds by sex and ethnicity, New Zealand Census Mortality Study.

Figure 4 shows the age-standardised mortality rate differences disaggregated by the major tobacco-related causes of mortality (see Supplementary Table S1 for SRD and SRR figures). The IHD smoking SRDs (the height of the IHD labelled part of the total bar) narrowed over time among European/Other men and women. Conversely, the IHD SRDs increased over time among Māori. The stroke SRDs were similar to IHD for European/Other, but Māori stroke SRDs were measured with considerable imprecision (Supplementary Table S1). SRDs for lung cancer and COPD were consistently high over time in Māori and European/Other men compared to women, with the highest SRDs in Māori men. In Māori and European/Other women, SRDs doubled for lung cancer (from 44 to 90 per 100,000 in European/Other and 79 to 164 in Māori) and for COPD. The difference between men and women reflects the generally increasing lung cancer and COPD mortality in female smokers and the generally stable lung cancer and COPD mortality rates in male smokers (Fig. 2).

### Confounding over time

Table 1 presents Poisson regression RRs comparing mortality in current- and never-smokers by sex for each of the three time periods, with sequential adjustment for confounders. There is a moderate reduction in the RR after adjustment for SEP and HHS at any point in time, but this confounding increases over time such that by 2006–11 among men the (excess) RRs reduce by 36% from 2.24 to 1.79 when additionally adjusted for SEP and HHS; the reduction was 24% for men in 1981-84 (RRs 1.63 adjusted for age and ethnicity, and 1.48 fully-adjusted). For women, the reduction due to confounding was 31% in 2006–11 compared to 15% in 1981–84.

**Table 1:** Poisson regression rate ratios for the association between smoking and mortality over time in three cohorts 25–74 years old in the New Zealand Census Mortality Study (95% confidence intervals in brackets).

	Rate ratios^a^		Men			Women		
			1981–84	1996–99	2006–11	1981–84	1996–99	2006–11
Age & ethnicity adjusted	All-cause, ethnicity-combined	RR	1.63 (1.55–1.72)	2.12 (2.02–2.23)	2.24 (2.14–2.33)	1.60 (1.51–1.69)	2.08 (1.96–2.20)	2.30﻿﻿ (2.19–2.42)
		% change in excess RR	—	75%	95%	—	80%	117%
Age, ethnicity & SEP adjusted	All-cause, ethnicity -combine	RR	1.51 (1.44–1.59)	1.82 (1.73–1.91)	1.85 (1.77–1.93)	1.54 (1.45–1.63)	1.87 (1.77–1.99)	1.97 (1.87–2.07)
		% change in excess RR	—	61%	67%	—	61%	80%
Age, ethnicity, SEP & HHS adjusted	All-cause, ethnicity -combined	RR	1.48 (1.40–1.56)	1.77 (1.68–1.87)	1.79 (1.71–1.87)	1.51 (1.42–1.61)	1.80 (1.69–1.92)	1.90 (1.80–2.00)
		% change in excess RR	—	60%	65%	—	57%	76%
	All-cause, by ethnicity^b^	Māori	0.97 (0.81–1.17)	1.35 (1.17–1.54)	1.28 (1.14–1.44)	1.10 (0.88–1.37)	1.17 (1.00–1.37)	1.38 (1.21–1.58)
		European/ Other	1.55 (1.47–1.64)	1.91 (1.80–2.03)	2.01 (1.91–2.11)	1.59 (1.50–1.70)	2.02 (1.89–2.16)	2.14 (2.02–2.27)
	Ethnicity-combined, by cause of death	IHD	1.42 (1.30–1.55)	1.75 (1.58–1.93)	2.03 (1.83–2.25)	1.86 (1.65–2.08)	2.38 (2.03–2.78)	2.90 (2.44–3.44)
		Stroke	1.59 (1.30–1.95)	1.53 (1.20–1.96)	1.76 (1.39–2.23)	1.73 (1.43–2.09)	2.41 (1.89–3.07)	2.75 (2.20–3.44)
		Lung cancer	7.84 (5.87–10.5)	9.83 (7.72–12.5)	9.71 (8.11–11.6)	8.73 (6.16–12.4)	10.0 (7.88–12.8)	11.0 (9.16–13.2)
		COPD	4.30 (3.00–6.16)	6.70 (4.76–9.43)	6.71 (4.98–9.02)	4.74 (3.19–7.03)	10.1 (7.15–14.2)	11.0 (8.28–14.5)

Notes: RR: rate ratio, SEP: socioeconomic position, HHS: household smoking, IHD: ischaemic heart disease, COPD: chronic obstructive pulmonary disease. a. Rate ratios are confined to the population with no missing socioeconomic position variables and no missing household smoking. b. In the fully-adjusted model for all-cause mortality the interaction of smoking and ethnicity was statistically significant (p-value < 0.0001) in each of the cohorts for both men and women.

### Changing smoking rate ratios over time by sex, cause of death, and ethnicity

Poisson smoking-mortality RR estimates fully-adjusted for SEP and HHS increased over time, mostly between 1981–84 and 1996–99 in men and women, with only modest increases from 1996–99 to 2006–11. A similar pattern was evident for lung cancer and COPD RRs (Table 1, Supplementary Figure S7) with relatively steeper increases in the RRs for IHD and stroke in women over time.

In the fully-adjusted Poisson model, there was an interaction between smoking and ethnicity with greater smoking-mortality RRs (p-value < 0.001) in European/Other than Māori at all time points for both sexes (Table 1). RRs increased in all sex-ethnicity groups.

## Discussion

This study indicates an increase in smoking-mortality RRs over time with differential phasing by social group (sex and ethnicity) and cause of death. Mortality declined over time in all groups of never-smokers; however, mortality trends in current-smokers varied by sex, ethnicity and cause of death (Fig. 2). Among European/Other, all-cause mortality rates declined in male smokers but were more stable in women smokers, consistent with findings in a US study^8^.

Patterns of results can be aligned and contrasted with the framework presented in Fig. 1 as an example of the phases of the tobacco epidemic. All-cause mortality SRDs for Māori and European/Other men increased from the 1980s to 1990s and then decreased in the 2000s; whereas for European/Other women the absolute mortality gap between current and never smokers appeared to plateau in the 1990s and 2000s, and among Māori women they increased over all decades. These results are consistent with the delayed peak in the tobacco epidemic intensity for women (Fig. 1). There is more variability again by cause of death. COPD/lung cancer SRDs increased among women but not men. Conversely, the SRD for COPD increased in 55 + year old men in a US study^8^ perhaps consistent with New Zealand up to 2011 being a step further along the framework timeline. IHD and stroke SRDs declined among European/Other in New Zealand (Fig. 4), but among Māori the IHD SRD increased over time. This likely indicates that Māori are both earlier in the tobacco epidemic and have experienced slower declines in never-smoker IHD mortality rates (Fig. 2).

The relative mortality gap between current and never (RRs) increased for men and women from 1981–84 to 1996–99 – with modest increases from 1996–99 to 2006–11 (except for Māori women where the increase was greater). This increasing relative mortality gap between current and never smokers occurred even after adjusting for an increase in the amount of confounding by SEP and HHS over time. Many long-term studies report increasing RRs over time^7–10^, however, the possible plateauing recently in our New Zealand study appears to be novel.

There are three likely contributing reasons for changing RRs over time: 1) the changing intensity of smoking over time, 2) decreasing mortality rates in never-smokers and 3) the time-lag from smoking to peak mortality harm. We expand these below in general terms that should apply to most countries, and point to examples from New Zealand as a case study.

1. Changes in smoking intensity over time
  Increased mortality in smokers is linked to smoking intensity or pack-year history^8, 9, 18^, including greater duration of smoking, age at which smoking was initiated, more cigarettes per day, increased inhalation per cigarette (eg, smoking down to the butt) and/or changes to the harmfulness of the product over time such as tar/carcinogen content or roll-your-own smoking. Using New Zealand as a case example, youth smoking prevalence in New Zealand was high in the 1960s and 1970s (and possibly earlier) for men and peaked in the mid-1970s for women^19^, consistent with a delayed phasing of the tobacco epidemic in women. Smoking prevalence in young Māori women remained at higher rates than in European/Other and decreased more slowly from the 1970s-1990s^19^.
2. Decreasing mortality rates in never-smokers
  All-cause mortality has declined substantially in never-smokers. Similar absolute mortality rate declines in current and never-smokers mathematically will – necessarily – result in an increase in the smoking-mortality RR. Furthermore, there is likely to be heterogeneity between social groups with varying background (and among never smokers) mortality rates due to differential timing of epidemiological transitions. Using New Zealand as a case example, Māori never-smokers are a population with high background mortality rates, due to other risk factors for mortality such as obesity, infectious diseases, and lower access to some health services^20^. If absolute excess mortality from smoking is fixed then higher never-smoker mortality will result in lower smoking-mortality RRs, as seen for Māori in this study. Our results are consistent with several studies internationally that report different smoking-mortality RR associations by social group including by ethnicity^15^, sex^8^ and country^21^.
3. Time-lag from smoking to peak mortality harm

Causes of death changed in their contribution to the excess smoking-mortality over time, consistent with heterogeneity by cause of death^8, 12, 22^. The different patterns between IHD/stroke and lung cancer/COPD are affected by the different time-lag between smoking and peak smoking-related mortality^14, 23^. Using New Zealand as a case example, in the early 1980s the excess lung cancer and COPD mortality in smokers (SRDs) was notably greater in men than women, but by the late 2000s SRDs in women had increased to be similar to men. This is consistent with historical differences in the phasing of the tobacco epidemic by sex (approximately 30 years is the time-lag from smoking to peak lung cancer mortality). However, throughout the study the IHD/stroke SRD trends by smoking status did not differ markedly by sex, consistent with similar effective doses of tobacco by sex in recent times and shorter time-lags to cardiovascular disease.

### Confounding

Smoking is increasingly concentrated in socially disadvantaged populations in many settings (eg, as per this European study^24^), and these populations usually have higher rates of other risk factors for non-communicable diseases. Thus, theoretically we expect confounding of the tobacco-mortality association to increase over time (eg, by socioeconomic factors, and more proximally diet, physical activity and alcohol consumption). This New Zealand case-study is consistent with that expectation, with increasing confounding of the smoking-mortality association by SEP over time. We were ‘only’ able to adjust for six socioeconomic factors, thus meaning residual confounding by risk factors (eg, diet, obesity) is possible. However, these risk factors are largely (not necessarily fully) explained by socioeconomic factors . In a previous paper we applied quantitative bias analysis methods to evaluate the marginal effect of confounding by alcohol and obesity (over and above SEP) on smoking-cancer associations, and showed that any residual confounding over and above SEP was likely to be small^25^. In this study we also adjusted for HHS, but compared to other SEP variables it was found to have little additional impact as a confounder of the active-smoking-mortality relationship.

### Study strengths and limitations

This New Zealand case-study included 16.1 million person-years of follow-up, three national cohort studies, high quality census data, a study timeframe representing 30 years and thorough adjustment for confounding by SEP. Sensitivity analyses suggest that selection bias (restriction to individuals with complete data in Poisson analysis) may have contributed a small degree to higher smoking RRs (Supplementary File). Mismeasurement bias and reverse causality from people quitting smoking due to illness may both have biased our results towards the null. In each cohort we had only one cross-sectional measure of an individual’s smoking status which does not allow for duration of smoking, cigarettes per day or type of tobacco consumed. Longitudinal examination of detailed smoking exposures would improve our understanding of the impact of dose on mortality. Nevertheless, trends in such biases are unlikely to change markedly over time (see Supplementary File) and thus they are unlikely to substantially impact on observed trends in RRs and RDs over time.

### Possible implications

These results signal the policy and research importance of understanding the phases of the tobacco epidemic, and the variation in mortality consequences from various trajectories of smoking prevalence. The heterogeneity in the smoking-mortality association over time has implications for how researchers project and quantify future health and inequality impacts of tobacco control interventions. Such improved information might allow policy-makers to more appropriately prioritise tobacco control interventions relative to competing ways to reduce health loss and health inequalities (eg, via obesity prevention interventions or enhanced alcohol control interventions). But of course such prioritisation also requires similar quality information on the other health problems (eg, the obesity epidemic) and also the health economic aspects of all the specific interventions (ie, the relative cost-effectiveness of specific interventions such as a tobacco tax vs a sugary drinks tax). If tobacco control interventions are prioritised, there are specific interventions that appear able to reduce inequalities in smoking (including tobacco tax increases^26^, some smoking cessation services^27^ and various “tobacco endgame strategies”^28^).

### Conclusions

This study reports an increase in the relative mortality gap between current and never smokers (RRs) over time and that this change persisted after adjusting for confounding by socioeconomic position. There was differential phasing by sex, ethnicity and cause of death. Variation in smoking-mortality RRs is likely to be related to changes in the intensity of smoking over time, underlying trends in mortality rates among never-smokers and time-lags between smoking and peak-mortality for different causes of death. Application of a framework helped provide understanding about the phasing of the tobacco epidemic. These findings have implications for estimating the future health and inequality impacts of tobacco control interventions and how these might be prioritised relative to other interventions that reduce health loss and reduce health inequalities.

## Methods

### Study population

Three closed cohorts of the New Zealand resident population living in a private dwelling, on census night in 1981, 1996 and 2006 were created by linking census and mortality records for three subsequent years following the 1981 and 1996 censuses and five years subsequent to the 2006 census. Probabilistic linkage was done with QualityStage TM software using an individual’s address (meshblock or census area unit), sex, date of birth, ethnicity and country of birth as matching variables. Approximately 98% of links were estimated to be true links where the mortality record was correctly linked to a previous census record^29^. This provided 111,000 deaths from 16.1 million person-years of follow-up (Table 2). The percentage of deaths linked to a census record ranged from 71% in 1981 to 83% in 2006. Linkage weights were used to adjust for incomplete linkage of mortality records to make the data representative of all deaths. Namely, the inverse probabilities of a mortality record being linked to the census in each age-ethnicity-deprivation-region strata were used as weights in the analysis. We present results for individuals aged 25–74 years old in the follow-up period, given more optimal measures of SEP in this group.

**Table 2:** Baseline characteristics of the study population by sex and years of follow-up for New Zealand 25–74 year olds in 1981, 1996, and 2006.

		Men			Women		
		1981–84	1996–99	2006–11	1981–84	1996–99	2006–11
Participants^**a**^	n (total)	855,000	995,400	1,095,900	882,800	1,059,900	1,188,400
	n (complete data)	627,500	784,500	865,900	637,800	834,800	937,400
	Person-years (complete)	1,659,700	2,139,200	4,001,600	1,691,500	2,279,300	4,355,100
Deaths^**b**^ (n)	All-cause	21,723	18,807	26,376	13,407	12,048	18,750
	IHD	7,815	4,920	5,091	3,441	1,764	1,740
	Stroke	1,461	828	996	1,311	717	936
	Lung cancer	1,851	1,575	2,292	615	1,005	1,983
	COPD	1,077	813	1,050	426	633	1,140
Smoking Status (%)	Smoker	35.1	24.0	20.4	29.3	24.0	18.1
	Ex-smoker	26.4	26.1	24.1	15.0	26.1	20.1
	Never smoker	37.0	45.8	47.5	54.0	45.8	53.9
	Missing	1.5	4.1	8.0	1.7	4.1	7.8
Household Smoking (%)	No	46.9	56.0	57.7	50.7	58.3	60.0
	Yes	51.3	37.7	31.8	47.4	35.3	29.8
	Missing	1.8	6.4	10.5	1.9	6.4	10.3
Age-group (%)	25–44 years	50.4	48.3	41.4	49.3	48.5	41.7
	45–64 years	34.4	35.6	42.7	32.6	33.7	41.6
	65–74 years	13.7	13.8	12.8	15.5	14.0	12.3
	Missing	1.5	2.4	3.1	2.7	3.8	4.4
Ethnicity (%)	Māori	8.5	10.9	10.2	8.6	11.3	11.0
	Pacific	2.5	3.9	4.5	2.4	4.0	4.6
	Asian	1.2	4.1	8.3	1.1	4.4	8.8
	European/Other	86.8	80.1	72.8	86.5	79.3	71.8
	Missing	1.0	1.0	4.2	1.4	1.0	3.8
Education (%)	No qualifications	47.4	32.0	21.9	52.7	34.5	21.6
	School qualification	17.0	26.2	26.7	18.1	31.8	31.4
	Post-school qualification	26.7	40.4	41.9	18.1	32.1	37.3
	Missing	8.9	1.5	9.4	11.0	1.6	9.8
NZDep Quintile (%)	Most deprived	19.3	22.2	21.3	19.2	21.6	20.7
	Second most deprived	20.7	21.0	20.9	20.3	20.6	20.6
	Average deprivation	20.6	20.3	20.3	20.5	20.2	20.3
	Second least deprived	20.3	19.3	19.7	20.4	19.6	20.1
	Least deprived	19.0	17.1	17.8	19.6	17.9	18.3
	Missing	0.1	0.1	0.0	0.1	0.1	0.0
Household Income (NZD)	Mean (CPI adjusted)^c^	16,400	44,600	51,900	14,800	40,700	47,900
	Standard deviation^c^	10,500	31,200	34,400	10,000	29,800	33,500
	Missing (%)	16.9	13.3	16.7	16.5	14.0	17.0
Labour Force Status (%)	Employed	80.6	71.2	72.6	45.6	54.7	59.6
	Unemployed	2.4	4.6	2.6	1.3	4.1	2.8
	Inactive	17.0	24.2	21.5	53.1	41.2	34.4
	Missing	—	0	3.4	—	0	3.1
Car Access (%)	Nil	8.1	6.4	4.1	12.3	10.3	6.5
	One	51.5	35.8	27.5	51.6	38.4	32.0
	Two or more	35.1	55.9	64.3	29.8	49.3	57.6
	Missing	5.3	1.8	4.1	6.4	2.0	3.9
Housing Tenure (%)	Owned	73.2	71.4	64.6	74.0	71.4	64.5
	Rented	26.8	23.5	29.7	26.0	23.8	29.8
	Not stated	—	5.2	5.7	—	4.8	5.7

Notes: IHD: Ischaemic heart disease, COPD: Chronic obstructive pulmonary disease. a. Usual residents living in a private dwelling on census night, b. Numbers are random rounded to base three to protect confidentiality, c. Income is consumer price index (CPI) adjusted to 1996 New Zealand Dollars (NZD). European/Other is defined as individuals who do not identify as Māori, Pacific or Asian, the large majority of whom were of the New Zealand European group.

Smoking information from the census night was categorised as current-smoker, ex-smoker, or never-smoker with ex-smokers put aside for the majority of analyses in this paper (see census questions in Supplementary Material Appendix B). HHS was defined as living in a household with at least one other person who currently smoked, irrespective of whether the index individual was a smoker or not. The outcomes were all-cause mortality and the four largest contributors to smoking-related mortality: lung cancer, ischaemic heart disease (IHD), stroke and chronic obstructive pulmonary disease (COPD). Ethnicity was self-identified and prioritised as Māori, Pacific, Asian or European/Other. The latter group comprised individuals who did not identify as Māori, Pacific or Asian, the large majority of whom were New Zealand European.

### Analysis

Age-standardised rates, standardised rate differences (SRDs) and standardised rate ratios (SRRs) were calculated to compare mortality in current-smokers with mortality in never-smokers. Direct standardisation was applied using the WHO World Standard Population to maximise international comparability.

Poisson regression was carried out in SAS^30^ to estimate the mortality rate ratio (RR) in current-smokers compared with never-smokers. The fully-adjusted model included age (5-year age-groups), ethnicity (prioritised by Māori, Pacific, Asian and European/Other), equivalised household income (using a New Zealand-specific index)^31^, neighbourhood deprivation quintile^32, 33^, education (none, school, post-school qualifications), labour force status (employed, job-searching, inactive), housing tenure (owner, renting, unknown), car access (0, 1 or 2+ motor vehicles) and HHS (none, lives with a current-smoker).

Sensitivity analyses were done to investigate the impact of selection bias (restricting to individuals with complete income/SEP information), reverse causation (by excluding first year of census-mortality cohort follow-up) and a negative control (smoking is expected to have a minimal association with unintentional injury). Results from sensitivity analyses did not raise major concerns about the main results presented in this paper (see Supplementary Material Appendix D).

### Data availability

Supplemental information with additional methods, results and further explanation of the smoking mortality relationship and phasing of the tobacco epidemic over time is attached. Programming code and data sharing is available on request from the authors.

### Ethical approval

Ethics approval was provided by the Central Regional Ethics Committee, reference number WGT/04/10/093.

## Electronic supplementary material

Supplementary Information
