# Supplementary material for: Changing smoking-mortality association over time and across social groups: National census-mortality cohort studies from 1981 to 2011
Source: Sci Rep. 2017 Sep 13;7:11465. doi: 10.1038/s41598-017-11785-x (PMC5597615; doi:10.1038/s41598-017-11785-x)
Supplement: Supplementary file 1 — Supplementary Information [file 41598_2017_11785_MOESM1_ESM.doc]

# Supplementary File

This Supplementary Material provides additional information to that in the article by Andrea Teng, June Atkinson, George Disney, Nick Wilson and Tony Blakely titled “Changing smoking-mortality association over time and across social groups: National census-mortality cohort studies from 1981 to 2011”.

Table of Contents

[Appendix A: The Smoking-Mortality Relationship and Phasing of the Tobacco Epidemic 2](#__RefHeading___Toc467758967)

[Appendix B: Additional Methods 4](#__RefHeading___Toc467758968)

[Smoking Related Questions used in Censuses (New Zealand) 4](#__RefHeading___Toc467758969)

[Household smoking 4](#__RefHeading___Toc467758970)

[Appendix C: Additional Results, Tables and Figures 5](#__RefHeading___Toc467758971)

[Standardised mortality rates 5](#__RefHeading___Toc467758972)

[Poisson regression 13](#__RefHeading___Toc467758973)

[Appendix D: Sensitivity Tests 20](#__RefHeading___Toc467758974)

# Appendix A: The Smoking-Mortality Relationship and Phasing of the Tobacco Epidemic

Figure S1 shows a stylistic possibility of phased hypothetical tobacco epidemics for three scenarios: a) long time-lags, and ‘low’ background mortality rates among never smokers; b) long time-lags and ‘high’ background mortality rates among never smokers; and short time-lags and low mortality rates among never smokers. Parameterization is detailed in the footnotes to Figure. In this example it takes 50 years for the maximum absolute impact (i.e. rate difference) and relative differences (rate ratio) in mortality between current and never smokers to be realised. The rate ratio keeps increasing over time within each scenario. Most epidemiological studies are a cross-section of this time varying situation, or a short window at best. In our New Zealand study, with cohorts from 1981-84 to 2006-11, a wider window of observation is used, within which we a priori expect: varying rate differences and rate ratios over time within each population group (e.g. the four sex by ethnic groups); and variation between population groups at each point in time.

Figure S1: Hypothetical progression of various tobacco epidemics over 100 years, for three scenarios: a) long time-lags b) long-time-lags and ‘high’ base mortality rate (BMR); c) short time-lags and ‘low’ BMR

Rate difference and rate ratio comparing current to never smokers;

|  | a) Long time-lags and ‘low’ BMR | b) long-time-lags and ‘high’ BMR | c) short time-lags and ‘low’ BMR |
| --- | --- | --- | --- |
| Mortality rates | 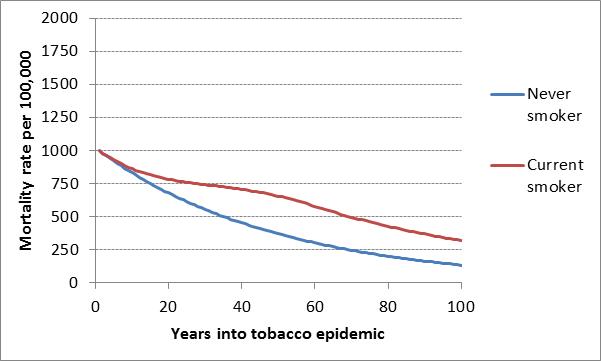 | 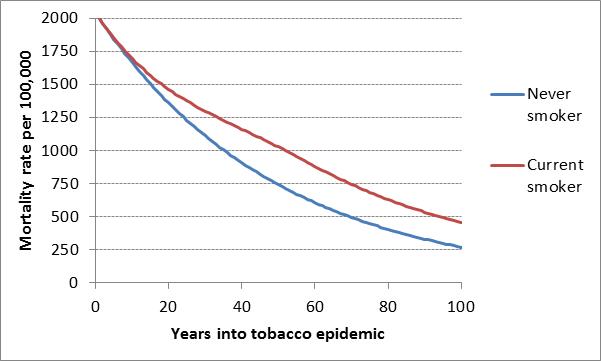 | 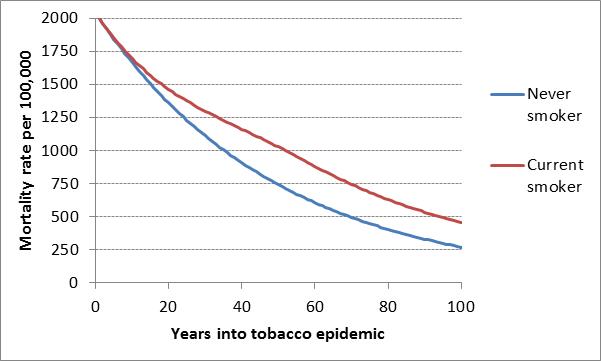 |
| Mortality rate differences (RDs) and rate ratios (RRs) | 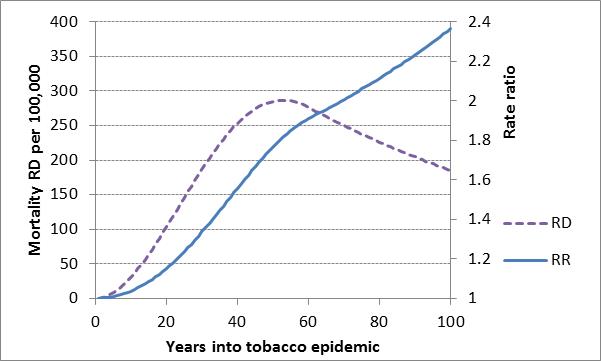 | 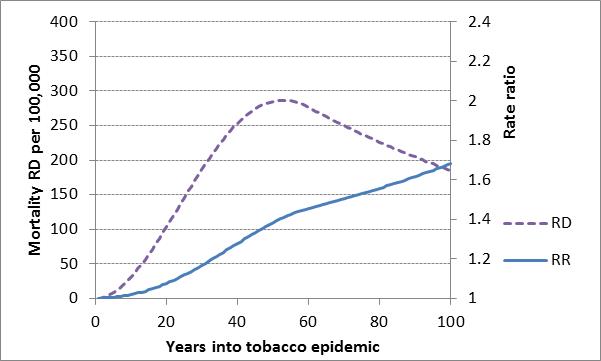 | 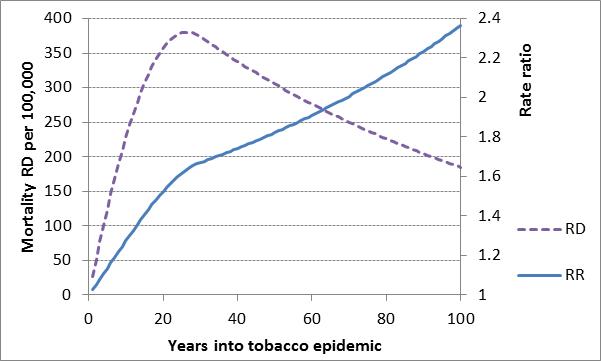 |

Starting mortality rates at year 0 for both current and never smokers = 1000 per 100,000 in ‘low’ BMR scenarios (a and c) and 2000 per 100,000 in ‘high’ BMR scenario. The BMR falls at 2% per annum. In the long time-lags scenario, peak excess mortality (on an absolute scale) for current versus never smokers is achieved at 60 years, and in short time-lag scenarios at 30 years. The peak absolute mortality rate difference between never and current smokers is 500 per 100,000 at year 0, reducing by 1% per annum. The functional form for the increasing gap between never and current smokers is a cosine (long time-lags) or sine (short time-lags) function.

# Appendix B: Additional Methods

## Smoking Related Questions used in Censuses (New Zealand)

In 1981, the census included the following question: (Easton, 1995)

“Cigarette Smoking: Tick the box which best describes your current cigarette smoking?

- Never smoked cigarettes at all, or never smoked them regularly.
- Do not smoke cigarettes now, but used to smoke them regularly (1 or more a day).
- Currently smoke cigarettes regularly (1 or more a day).”

The question treats those who smoke, and have only smoked, cigars and pipe tobacco as non-smokers of cigarettes. Chewing tobacco and snuff is extremely uncommon in New Zealand. Sales of loose tobacco exceeded manufactured cigarettes (by weight) until 1955. But loose tobacco is used for roll-your-owns as well as pipes, and many pipe smokers may also have used cigarettes on a regular basis at some stage. Pipe smoking has been fairly rare in New Zealand in recent decades. (Easton, 1995)

In 1996 and 2006 the census questions were: (Statistics New Zealand)

- “Do you smoke cigarettes regularly (that is, one or more a day)? Don’t count pipes, cigars or cigarillos. Count only tobacco cigarettes. Yes No”
- “Have you even been a regular smoker of one or more cigarettes a day? Yes No”

EASTON, B. 1995. Smoking in New Zealand: a Census Investigation*. Australian Journal of Public Healt*h, **1**9, 125-129.

## Household smoking

We use the HHS variable as a proxy for passive smoking, to adjust for HHS confounding of the association of active smoking with mortality over time. Fewer never-smokers are exposed to passive smoking over time which may reduce the mortality in this group. HHS information could not be obtained if an individual lived with one or more adults for which smoking data was unavailable (unless one or more other adults in the household were already known to be a smoker).

# Appendix C: Additional Results, Tables and Figures

## Standardised mortality rates

Table S1: Age and ethnicity standardised mortality rates (per 100,000), rate differences and rate ratios with the corresponding 95% confidence intervals by cause of death.

|  |  | Never Smoked | Smoker |  |  |  |  |  |  | Ex-Smoker |  |  |  |  |  |  |
| --- | --- | --- | --- | --- | --- | --- | --- | --- | --- | --- | --- | --- | --- | --- | --- | --- |
|  |  | Rate | Rate | SRD | (lower- | upper) | SRR | (lower- | upper) | Rate | SRD | (lower- | upper) | SRR | (lower- | upper) |
| **Men** |  |  |  |  |  |  |  |  |  |  |  |  |  |  |  |  |
| IHD | 1981-84 | 256.4 | 369.6 | 113 | 87 | 139 | 1.44 | 1.32 | 1.57 | 320.2 | 64 | 37 | 91 | 1.25 | 1.14 | 1.37 |
|  | 1996-99 | 141.1 | 257.6 | 117 | 98 | 135 | 1.83 | 1.66 | 2.00 | 173.2 | 32 | 17 | 47 | 1.23 | 1.12 | 1.35 |
|  | 2006-11 | 71.5 | 164.4 | 93 | 81 | 105 | 2.30 | 2.09 | 2.53 | 98.4 | 27 | 19 | 35 | 1.38 | 1.25 | 1.51 |
| Stroke | 1981-84 | 58.0 | 76.2 | 18 | 5 | 32 | 1.31 | 1.06 | 1.62 | 49.8 | -8 | -21 | 4 | 0.86 | 0.68 | 1.08 |
|  | 1996-99 | 29.3 | 48.4 | 19 | 10 | 28 | 1.65 | 1.32 | 2.08 | 22.6 | -7 | -13 | -0 | 0.77 | 0.60 | 0.99 |
|  | 2006-11 | 17.2 | 26.7 | 10 | 4 | 15 | 1.55 | 1.25 | 1.94 | 20.8 | 4 | -0 | 8 | 1.21 | 0.98 | 1.48 |
| Lung cancer | 1981-84 | 17.5 | 138.6 | 121 | 108 | 135 | 7.93 | 5.88 | 10.69 | 66.3 | 49 | 39 | 59 | 3.79 | 2.78 | 5.18 |
|  | 1996-99 | 17.1 | 145.9 | 129 | 115 | 142 | 8.54 | 6.74 | 10.82 | 58.7 | 42 | 34 | 50 | 3.44 | 2.68 | 4.42 |
|  | 2006-11 | 12.7 | 136.1 | 124 | 113 | 134 | 10.74 | 9.03 | 12.79 | 47.0 | 34 | 29 | 39 | 3.71 | 3.08 | 4.46 |
| COPD | 1981-84 | 15.3 | 65.8 | 51 | 40 | 61 | 4.29 | 2.92 | 6.31 | 51.9 | 37 | 24 | 49 | 3.39 | 2.21 | 5.18 |
|  | 1996-99 | 8.1 | 68.0 | 60 | 50 | 70 | 8.41 | 5.95 | 11.90 | 36.3 | 28 | 22 | 35 | 4.49 | 3.14 | 6.43 |
|  | 2006-11 | 7.0 | 57.1 | 50 | 43 | 58 | 8.19 | 6.21 | 10.79 | 28.1 | 21 | 17 | 25 | 4.02 | 3.04 | 5.32 |
| Unintentional injury | 1981-84 | 55.1 | 68.5 | 13 | -0 | 27 | 1.24 | 0.99 | 1.56 | 42.5 | -13 | -27 | 1 | 0.77 | 0.57 | 1.04 |
|  | 1996-99 | 35.7 | 47.5 | 12 | 2 | 21 | 1.33 | 1.06 | 1.66 | 42.3 | 7 | -6 | 19 | 1.18 | 0.87 | 1.61 |
|  | 2006-11 | 26.6 | 46.2 | 20 | 14 | 26 | 1.74 | 1.48 | 2.04 | 33.2 | 7 | 0 | 13 | 1.25 | 1.02 | 1.53 |
| Suicide | 1981-84 | 19.8 | 25.7 | 6 | -1 | 13 | 1.30 | 0.96 | 1.77 | 24.0 | 4 | -7 | 16 | 1.21 | 0.74 | 2.00 |
|  | 1996-99 | 20.0 | 56.8 | 37 | 28 | 46 | 2.85 | 2.25 | 3.61 | 25.9 | 6 | -3 | 15 | 1.30 | 0.91 | 1.84 |
|  | 2006-11 | 16.4 | 39.6 | 23 | 18 | 28 | 2.41 | 2.01 | 2.88 | 20.9 | 5 | -2 | 11 | 1.27 | 0.94 | 1.72 |
| **Women** |  |  |  |  |  |  |  |  |  |  |  |  |  |  |  |  |
| IHD | 1981-84 | 104.8 | 186.4 | 82 | 62 | 101 | 1.78 | 1.57 | 2.02 | 145.6 | 41 | 20 | 62 | 1.39 | 1.19 | 1.62 |
|  | 1996-99 | 45.5 | 108.1 | 63 | 50 | 75 | 2.37 | 2.04 | 2.75 | 73.5 | 28 | 17 | 39 | 1.61 | 1.37 | 1.90 |
|  | 2006-11 | 22.1 | 70.4 | 48 | 40 | 56 | 3.18 | 2.73 | 3.71 | 39.7 | 18 | 12 | 23 | 1.79 | 1.52 | 2.12 |
| Stroke | 1981-84 | 44.7 | 69.3 | 25 | 13 | 36 | 1.55 | 1.27 | 1.89 | 56.0 | 11 | -4 | 26 | 1.25 | 0.95 | 1.66 |
|  | 1996-99 | 18.8 | 44.0 | 25 | 17 | 34 | 2.33 | 1.84 | 2.97 | 25.2 | 6 | -0 | 13 | 1.34 | 1.01 | 1.77 |
|  | 2006-11 | 13.0 | 31.3 | 18 | 13 | 23 | 2.40 | 1.96 | 2.94 | 15.7 | 3 | -1 | 6 | 1.21 | 0.95 | 1.53 |
| Lung cancer | 1981-84 | 10.1 | 57.3 | 47 | 38 | 57 | 5.65 | 3.74 | 8.55 | 42.1 | 32 | 20 | 44 | 4.15 | 2.59 | 6.65 |
|  | 1996-99 | 12.5 | 97.4 | 85 | 73 | 97 | 7.81 | 6.16 | 9.89 | 53.6 | 41 | 32 | 50 | 4.29 | 3.29 | 5.60 |
|  | 2006-11 | 11.1 | 107.9 | 97 | 88 | 106 | 9.76 | 8.25 | 11.55 | 42.5 | 32 | 27 | 36 | 3.85 | 3.22 | 4.59 |
| COPD | 1981-84 | 7.1 | 28.2 | 21 | 15 | 27 | 4.00 | 2.63 | 6.07 | 36.3 | 29 | 21 | 38 | 5.14 | 3.31 | 7.97 |
|  | 1996-99 | 6.5 | 55.8 | 49 | 41 | 58 | 8.57 | 6.06 | 12.13 | 40.1 | 34 | 27 | 41 | 6.16 | 4.33 | 8.75 |
|  | 2006-11 | 6.2 | 65.6 | 60 | 52 | 67 | 10.67 | 8.19 | 13.89 | 35.5 | 29 | 25 | 34 | 5.78 | 4.43 | 7.53 |
| Unintentional injury | 1981-84 | 13.5 | 18.9 | 5 | 0 | 11 | 1.40 | 1.02 | 1.92 | 16.3 | 3 | -4 | 9 | 1.21 | 0.80 | 1.82 |
|  | 1996-99 | 9.1 | 14.3 | 5 | 1 | 10 | 1.57 | 1.11 | 2.24 | 10.4 | 1 | -3 | 6 | 1.14 | 0.75 | 1.73 |
|  | 2006-11 | 9.5 | 16.6 | 7 | 4 | 10 | 1.74 | 1.38 | 2.20 | 9.7 | 0 | -2 | 3 | 1.02 | 0.79 | 1.31 |
| Suicide | 1981-84 | 6.4 | 11.7 | 5 | 2 | 9 | 1.84 | 1.19 | 2.85 | 6.2 | -0 | -4 | 4 | 0.98 | 0.52 | 1.84 |
|  | 1996-99 | 7.2 | 10.0 | 3 | -1 | 7 | 1.38 | 0.88 | 2.18 | 11.1 | 4 | -2 | 10 | 1.53 | 0.84 | 2.81 |
|  | 2006-11 | 4.6 | 15.1 | 11 | 7 | 14 | 3.31 | 2.43 | 4.51 | 4.0 | -1 | -2 | 1 | 0.89 | 0.61 | 1.29 |

Table S2: Age standardised mortality rates (per 100,000), rate differences and rate ratios among European/Other with the corresponding 95% confidence intervals by cause of death

|  |  | Never Smoked | Smoker |  |  |  |  |  |  | Ex-Smoker |  |  |  |  |  |  |
| --- | --- | --- | --- | --- | --- | --- | --- | --- | --- | --- | --- | --- | --- | --- | --- | --- |
|  |  | Rate | Rate | SRD | (lower- | upper) | SRR | (lower- | upper) | Rate | SRD | (lower- | upper) | SRR | (lower- | upper) |
| European/Other men | | |  |  |  |  |  |  |  |  |  |  |  |  |  |  |
| IHD | 1981-84 | 235.4 | 368.5 | 133 | 112 | 155 | 1.57 | 1.45 | 1.69 | 293.1 | 58 | 39 | 76 | 1.24 | 1.16 | 1.34 |
|  | 1996-99 | 104.5 | 235.3 | 131 | 113 | 148 | 2.25 | 2.04 | 2.48 | 133.3 | 29 | 19 | 39 | 1.28 | 1.17 | 1.39 |
|  | 2006-11 | 50.1 | 142.0 | 92 | 81 | 103 | 2.84 | 2.56 | 3.15 | 71.7 | 22 | 16 | 27 | 1.43 | 1.31 | 1.57 |
| Stroke | 1981-84 | 44.9 | 76.6 | 32 | 22 | 42 | 1.71 | 1.43 | 2.03 | 47.2 | 2 | -6 | 11 | 1.05 | 0.88 | 1.26 |
|  | 1996-99 | 18.9 | 45.1 | 26 | 18 | 34 | 2.39 | 1.89 | 3.02 | 19.6 | 1 | -3 | 5 | 1.04 | 0.84 | 1.29 |
|  | 2006-11 | 12.2 | 23.5 | 11 | 6 | 16 | 1.92 | 1.50 | 2.46 | 14.4 | 2 | -1 | 5 | 1.18 | 0.96 | 1.45 |
| Lung cancer | 1981-84 | 14.0 | 124.8 | 111 | 101 | 121 | 8.89 | 6.92 | 11.41 | 64.1 | 50 | 43 | 57 | 4.56 | 3.54 | 5.88 |
|  | 1996-99 | 9.3 | 118.5 | 109 | 98 | 121 | 12.68 | 9.94 | 16.18 | 43.8 | 35 | 30 | 39 | 4.69 | 3.68 | 5.98 |
|  | 2006-11 | 7.5 | 115.2 | 108 | 97 | 118 | 15.28 | 12.57 | 18.57 | 32.5 | 25 | 22 | 28 | 4.31 | 3.54 | 5.24 |
| COPD | 1981-84 | 10.7 | 59.9 | 49 | 41 | 57 | 5.60 | 4.12 | 7.62 | 49.0 | 38 | 32 | 44 | 4.58 | 3.39 | 6.20 |
|  | 1996-99 | 5.5 | 64.7 | 59 | 50 | 68 | 11.7 | 8.35 | 16.40 | 25.6 | 20 | 17 | 24 | 4.64 | 3.33 | 6.45 |
|  | 2006-11 | 3.2 | 52.8 | 50 | 42 | 57 | 16.4 | 12.03 | 22.36 | 18.4 | 15 | 13 | 18 | 5.72 | 4.23 | 7.74 |
| Unintentional injury | 1981-84 | 43.6 | 56.2 | 13 | 2 | 23 | 1.29 | 1.04 | 1.59 | 39.0 | -5 | -17 | 8 | 0.90 | 0.65 | 1.23 |
|  | 1996-99 | 30.7 | 42.2 | 12 | 1 | 22 | 1.38 | 1.05 | 1.80 | 37.9 | 7 | -7 | 22 | 1.24 | 0.83 | 1.84 |
|  | 2006-11 | 24.7 | 45.6 | 21 | 14 | 28 | 1.85 | 1.53 | 2.23 | 31.1 | 6 | -1 | 14 | 1.26 | 0.99 | 1.60 |
| Suicide | 1981-84 | 19.3 | 28.5 | 9 | 2 | 17 | 1.48 | 1.08 | 2.02 | 24.6 | 5 | -7 | 18 | 1.28 | 0.76 | 2.16 |
|  | 1996-99 |  |  |  |  |  |  |  |  |  |  |  |  |  |  |  |
|  | 2006-11 | 16.1 | 42.5 | 26 | 20 | 33 | 2.64 | 2.15 | 3.23 | 19.8 | 4 | -3 | 10 | 1.23 | 0.88 | 1.73 |
| European/Other women | | | |  |  |  |  |  |  |  |  |  |  |  |  |  |
| IHD | 1981-84 | 86.4 | 178.8 | 93 | 78 | 107 | 2.07 | 1.88 | 2.28 | 123.1 | 37 | 24 | 50 | 1.43 | 1.27 | 1.60 |
|  | 1996-99 | 29 | 91.3 | 62 | 51 | 73 | 3.15 | 2.7 | 3.66 | 53.1 | 24 | 18 | 31 | 1.83 | 1.58 | 2.11 |
|  | 2006-11 | 13.5 | 59.2 | 46 | 38 | 53 | 4.39 | 3.71 | 5.19 | 24.6 | 11 | 8 | 15 | 1.82 | 1.54 | 2.15 |
| Stroke | 1981-84 | 34.2 | 67.5 | 33 | 24 | 43 | 1.97 | 1.67 | 2.32 | 42.5 | 8 | 0 | 17 | 1.24 | 1.01 | 1.52 |
|  | 1996-99 | 11.5 | 40.8 | 29 | 22 | 37 | 3.55 | 2.82 | 4.47 | 18.8 | 7 | 3 | 11 | 1.63 | 1.28 | 2.08 |
|  | 2006-11 | 9.5 | 29.3 | 20 | 15 | 25 | 3.09 | 2.47 | 3.87 | 10.9 | 1 | -1 | 4 | 1.15 | 0.91 | 1.45 |
| Lung cancer | 1981-84 | 4.4 | 48.3 | 44 | 37 | 51 | 11.02 | 7.99 | 15.20 | 30.0 | 26 | 19 | 32 | 6.83 | 4.8 | 9.71 |
|  | 1996-99 | 6.8 | 85.1 | 78 | 68 | 89 | 12.51 | 9.84 | 15.91 | 33.4 | 27 | 22 | 32 | 4.92 | 3.82 | 6.32 |
|  | 2006-11 | 6.1 | 95.9 | 90 | 81 | 99 | 15.64 | 12.97 | 18.86 | 34.0 | 28 | 24 | 31 | 5.55 | 4.59 | 6.71 |
| COPD | 1981-84 | 3.7 | 26.8 | 23 | 18 | 28 | 7.27 | 5.08 | 10.41 | 28.7 | 25 | 19 | 31 | 7.78 | 5.39 | 11.23 |
|  | 1996-99 | 2.5 | 51.6 | 49 | 41 | 57 | 20.95 | 14.68 | 29.91 | 33.7 | 31 | 27 | 36 | 13.67 | 9.66 | 19.34 |
|  | 2006-11 | 2.9 | 61.1 | 58 | 50 | 66 | 21.31 | 16.21 | 28.02 | 27.6 | 25 | 21 | 28 | 9.62 | 7.37 | 12.56 |
| Unintentional injury | 1981-84 | 12.9 | 17.3 | 4 | -1 | 9 | 1.34 | 0.98 | 1.85 | 14.6 | 2 | -4 | 7 | 1.13 | 0.78 | 1.64 |
|  | 1996-99 | 7.6 | 12.9 | 5 | 1 | 10 | 1.71 | 1.14 | 2.57 | 8.3 | 1 | -2 | 4 | 1.09 | 0.74 | 1.62 |
|  | 2006-11 | 8.5 | 16.8 | 8 | 5 | 12 | 1.97 | 1.51 | 2.56 | 9.3 | 1 | -2 | 3 | 1.09 | 0.82 | 1.43 |
| Suicide | 1981-84 | 7.7 | 13.5 | 6 | 1 | 10 | 1.76 | 1.11 | 2.78 | 6.7 | -1 | -5 | 4 | 0.88 | 0.47 | 1.65 |
|  | 1996-99 |  |  |  |  |  |  |  |  |  |  |  |  |  |  |  |
|  | 2006-11 | 4.5 | 16.7 | 12 | 8 | 16 | 3.75 | 2.64 | 5.32 | 4.9 | 0 | -1 | 2 | 1.1 | 0.74 | 1.63 |

Table S3: Age standardised mortality rates (per 100,000), rate differences and rate ratios among Māori with the corresponding 95% confidence intervals by cause of death.

|  |  | Never Smoked | Smoker |  |  |  |  |  |  | Ex-Smoker |  |  |  |  |  |  |
| --- | --- | --- | --- | --- | --- | --- | --- | --- | --- | --- | --- | --- | --- | --- | --- | --- |
|  |  | Rate | Rate | SRD | (lower- | upper) | SRR | (lower- | upper) | Rate | SRD | (lower- | upper) | SRR | (lower- | upper) |
| **Māori men** | |  |  |  |  |  |  |  |  |  |  |  |  |  |  |  |
| IHD | 1981-84 | 440.6 | 435.0 | -6 | -132 | 121 | 0.99 | 0.74 | 1.32 | 440.2 | -1 | -135 | 134 | 1 | 0.74 | 1.36 |
|  | 1996-99 | 298.5 | 405.2 | 107 | 24 | 190 | 1.36 | 1.08 | 1.71 | 352.7 | 54 | -19 | 128 | 1.18 | 0.94 | 1.48 |
|  | 2006-11 | 173.3 | 306.9 | 134 | 76 | 191 | 1.77 | 1.42 | 2.21 | 242.0 | 69 | 28 | 110 | 1.40 | 1.15 | 1.70 |
| Stroke | 1981-84 | 105.1 | 58.4 | -47 | -103 | 10 | 0.56 | 0.29 | 1.06 | 74.4 | -31 | -95 | 34 | 0.71 | 0.34 | 1.45 |
|  | 1996-99 | 68.3 | 73.9 | 6 | -35 | 46 | 1.08 | 0.61 | 1.90 | 27.4 | -41 | -71 | -11 | 0.40 | 0.20 | 0.79 |
|  | 2006-11 | 33.1 | 37.2 | 4 | -15 | 23 | 1.12 | 0.65 | 1.93 | 41.7 | 9 | -10 | 27 | 1.26 | 0.77 | 2.05 |
| Lung cancer | 1981-84 | 32.6 | 247.2 | 215 | 141 | 288 | 7.58 | 3.13 | 18.34 | 93.8 | 61 | 8 | 114 | 2.88 | 1.09 | 7.57 |
|  | 1996-99 | 58.3 | 298.7 | 240 | 179 | 302 | 5.12 | 3.33 | 7.87 | 115.2 | 57 | 18 | 96 | 1.98 | 1.23 | 3.18 |
|  | 2006-11 | 26.6 | 257.4 | 231 | 183 | 278 | 9.67 | 6.42 | 14.55 | 107.2 | 81 | 56 | 105 | 4.02 | 2.64 | 6.13 |
| COPD | 1981-84 | 36.4 | 123.9 | 88 | 34 | 141 | 3.40 | 1.43 | 8.13 | 48.5 | 12 | -30 | 54 | 1.33 | 0.48 | 3.68 |
|  | 1996-99 | 16.8 | 106.1 | 89 | 47 | 131 | 6.32 | 2.78 | 14.38 | 76.4 | 60 | 29 | 91 | 4.56 | 2.01 | 10.32 |
|  | 2006-11 | 19.2 | 95.5 | 76 | 46 | 107 | 4.98 | 2.85 | 8.71 | 69.8 | 51 | 31 | 70 | 3.64 | 2.14 | 6.19 |
| Unintentional injury | 1981-84 | 123.1 | 122.8 | 0 | -68 | 67 | 1.00 | 0.58 | 1.73 | 78.1 | -45 | -111 | 21 | 0.63 | 0.33 | 1.22 |
|  | 1996-99 | 73.8 | 85.2 | 11 | -24 | 47 | 1.15 | 0.73 | 1.81 | 58.3 | -16 | -51 | 19 | 0.79 | 0.46 | 1.35 |
|  | 2006-11 | 55.1 | 70.9 | 16 | -6 | 38 | 1.29 | 0.91 | 1.83 | 57.4 | 2 | -23 | 27 | 1.04 | 0.67 | 1.62 |
| Suicide | 1981-84 | 27.2 | 18.4 | -9 | -33 | 15 | 0.67 | 0.25 | 1.85 | 10.9 | -16 | -42 | 9 | 0.40 | 0.08 | 1.94 |
|  | 1996-99 | . | . | . | . | . | . | . | . | . | . | . | . | . | . | . |
|  | 2006-11 | 21.1 | 44.5 | 23 | 9 | 38 | 2.11 | 1.31 | 3.38 | 33.4 | 12 | -12 | 36 | 1.58 | 0.72 | 3.44 |
| **Māori women** | |  |  |  |  |  |  |  |  |  |  |  |  |  |  |  |
| IHD | 1981-84 | 235.3 | 250.6 | 15 | -79 | 110 | 1.07 | 0.72 | 1.57 | 263.5 | 28 | -76 | 132 | 1.12 | 0.74 | 1.69 |
|  | 1996-99 | 124.1 | 205.9 | 82 | 28 | 136 | 1.66 | 1.21 | 2.27 | 179.3 | 55 | -1 | 111 | 1.44 | 1.02 | 2.05 |
|  | 2006-11 | 64.0 | 128.4 | 64 | 34 | 95 | 2.00 | 1.47 | 2.72 | 105.6 | 42 | 15 | 69 | 1.65 | 1.20 | 2.26 |
| Stroke | 1981-84 | 105.3 | 100.2 | -5 | -62 | 52 | 0.95 | 0.55 | 1.66 | 120.2 | 15 | -61 | 91 | 1.14 | 0.59 | 2.20 |
|  | 1996-99 | 46.2 | 68.2 | 22 | -18 | 62 | 1.48 | 0.78 | 2.81 | 59.0 | 13 | -22 | 47 | 1.28 | 0.68 | 2.39 |
|  | 2006-11 | 22.0 | 36.4 | 14 | 0 | 29 | 1.66 | 1.02 | 2.68 | 30.3 | 8 | -6 | 22 | 1.38 | 0.82 | 2.33 |
| Lung cancer | 1981-84 | 45.7 | 125.1 | 79 | 27 | 132 | 2.74 | 1.38 | 5.44 | 112.8 | 67 | -3 | 138 | 2.47 | 1.09 | 5.62 |
|  | 1996-99 | 38.7 | 178.8 | 140 | 94 | 186 | 4.62 | 2.90 | 7.35 | 159.9 | 121 | 70 | 172 | 4.13 | 2.51 | 6.79 |
|  | 2006-11 | 31.4 | 195.3 | 164 | 131 | 197 | 6.22 | 4.42 | 8.76 | 95.3 | 64 | 42 | 86 | 3.03 | 2.10 | 4.39 |
| COPD | 1981-84 | 26.7 | 47.6 | 21 | -7 | 49 | 1.78 | 0.81 | 3.92 | 90.5 | 64 | 14 | 114 | 3.39 | 1.49 | 7.73 |
|  | 1996-99 | 29.9 | 106.1 | 76 | 33 | 119 | 3.55 | 1.97 | 6.41 | 74.4 | 45 | 11 | 78 | 2.49 | 1.36 | 4.58 |
|  | 2006-11 | 26.1 | 101 | 75 | 47 | 103 | 3.86 | 2.48 | 6.03 | 84.5 | 58 | 35 | 81 | 3.23 | 2.09 | 5.01 |
| Unintentional injury | 1981-84 | 14.5 | 26.5 | 12 | -6 | 30 | 1.83 | 0.70 | 4.78 | 23.3 | 9 | -18 | 35 | 1.61 | 0.44 | 5.86 |
|  | 1996-99 | 16.0 | 29.2 | 13 | -4 | 31 | 1.83 | 0.85 | 3.91 | 10.6 | -5 | -19 | 8 | 0.66 | 0.23 | 1.89 |
|  | 2006-11 | 19.3 | 20.8 | 2 | -9 | 12 | 1.08 | 0.65 | 1.79 | 13.5 | -6 | -16 | 4 | 0.70 | 0.37 | 1.30 |
| Suicide | 1981-84 | . | 3.5 | 4 | -1 | 7 | . | . | . | 6.3 | 6 | -6 | 19 | . | . | . |
|  | 1996-99 | . | . | . | . | . | . | . | . | . | . | . | . | . | . | . |
|  | 2006-11 | 4.8 | 13.2 | 8 | 2 | 15 | 2.76 | 1.08 | 7.05 | 0.8 | -4 | -8 | 1 | 0.17 | 0.02 | 1.45 |

Figure S2: Age (and ethnicity) standardised mortality rates in men for multiple smoking-related causes of death over time by sex and ethnicity, 25-74 year olds, New Zealand Census Mortality Study
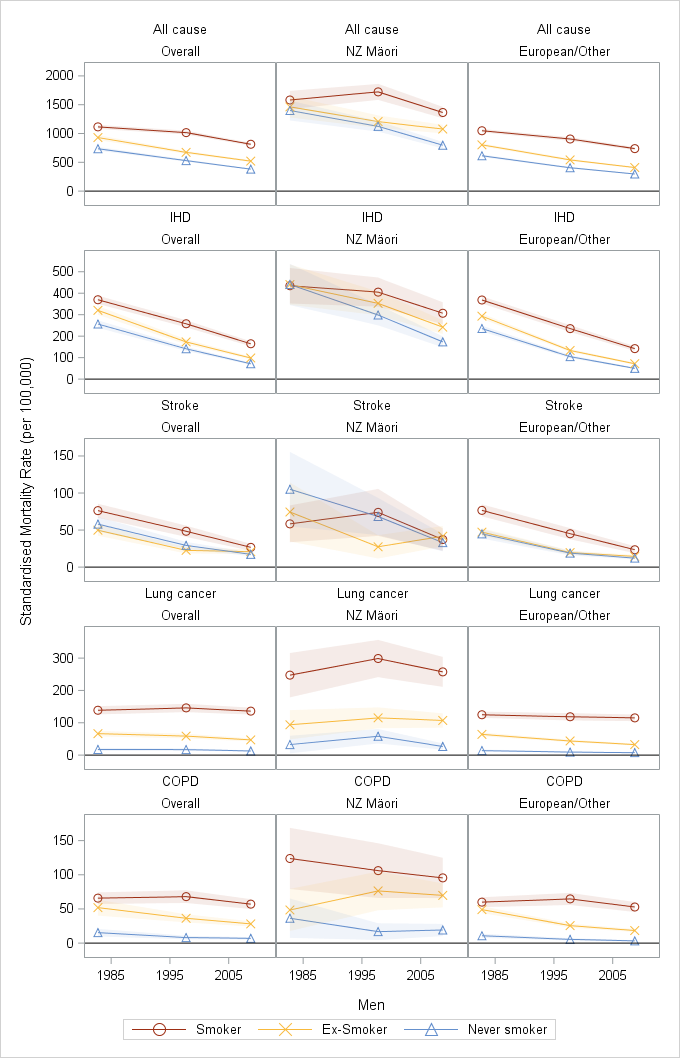


Figure S3: Age (and ethnicity) standardised mortality rates in women for multiple smoking-related causes of death over time by sex and ethnicity, 25-74 year olds, New Zealand Census Mortality Study
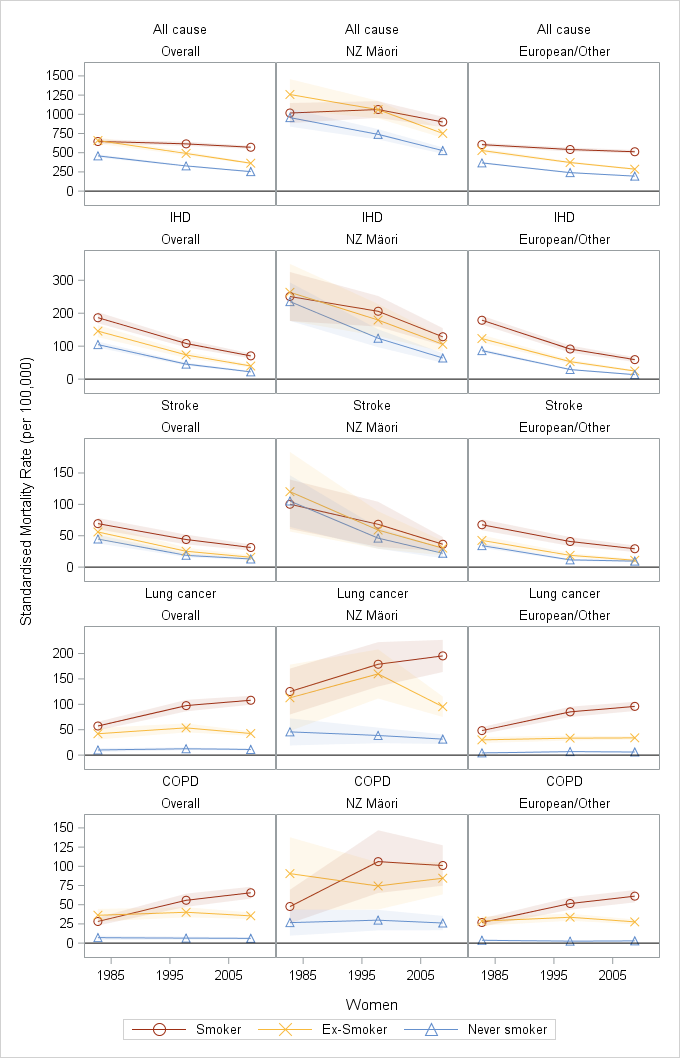


Figure S4: All-cause standardised mortality rates decomposed by mortality type for four major types of smoking-related causes of death


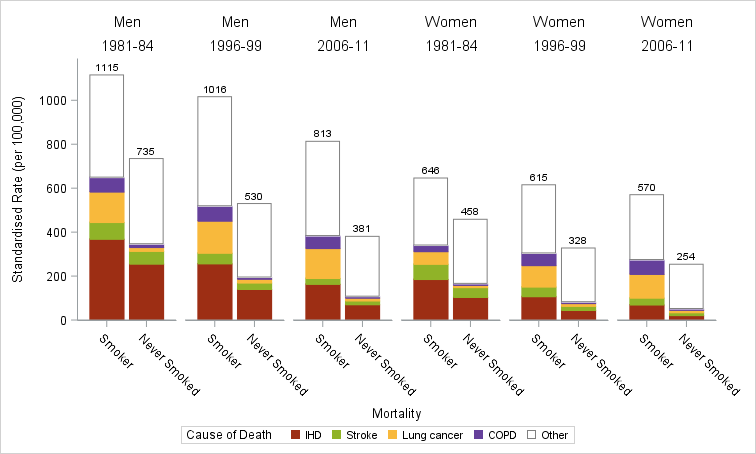


Figure S4: Age- and ethnicity- standardised rates ratios comparing current and never smokers over time by sex and cause of death, 25-74 year olds, New Zealand Census Mortality Study
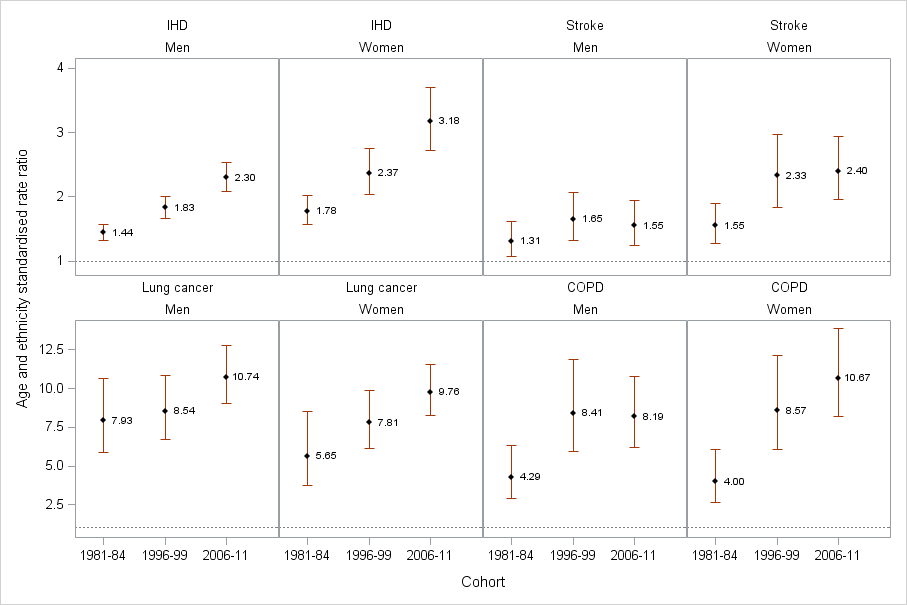


## Poisson regression

Figure S5: Poisson model estimates for the rate ratio comparing all-cause mortality in current and never smokers in three models with adjustment (adj) for 1) age and ethnicity (eth), 2) age, ethnicity and six variables for socioeconomic position (SEP) and 3) age, ethnicity, SEP and household smoking (HHS); for 25-74 year olds by sex over time, New Zealand Census Mortality Study. Note: study population confined to usual residents living in a private dwelling and present on census night, and those with complete data on all variables in the full model.


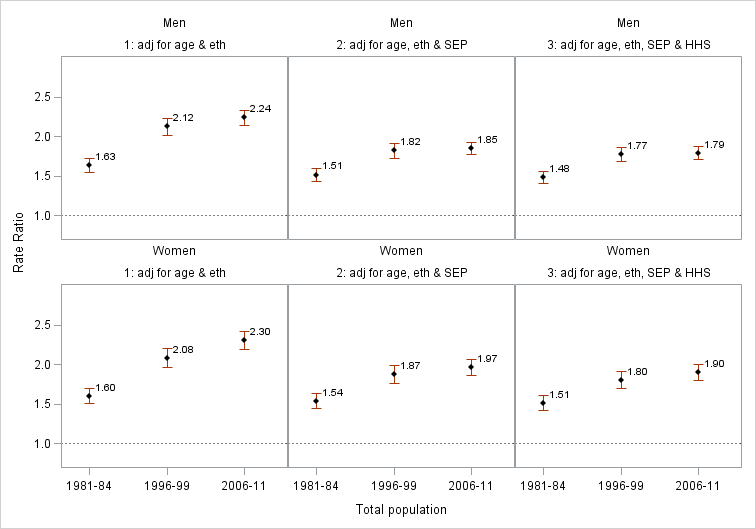


Figure S6: Poisson modelled smoking-mortality association by mortality type, sex and cohort for the total population. New Zealand Census Mortality Study 25-74 year olds. The rate ratio compares current-smokers to never-smokers with adjustment for age, sex, socioeconomic position and household smoking.


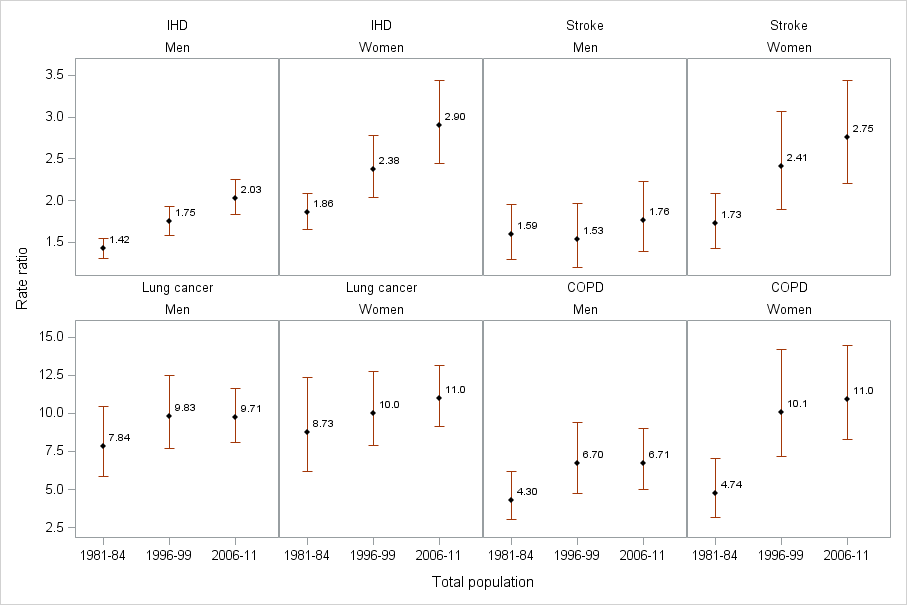


Figure S8: Poisson modelled smoking-mortality association by mortality type, sex and cohort for Māori and European/Other. New Zealand Census Mortality Study 25-74 year olds. The rate ratio compares current-smokers to never-smokers with adjustment for age, sex, socioeconomic position and household smoking.


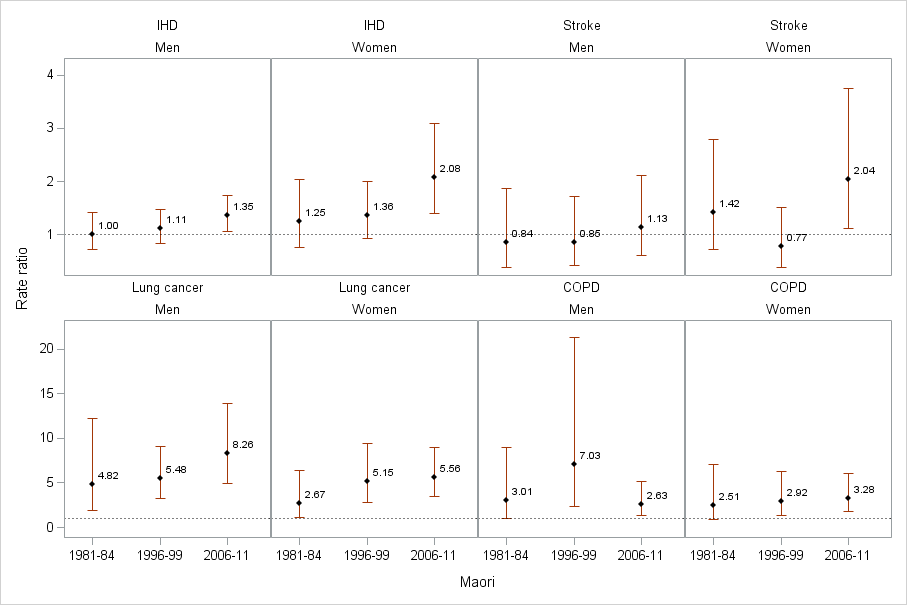


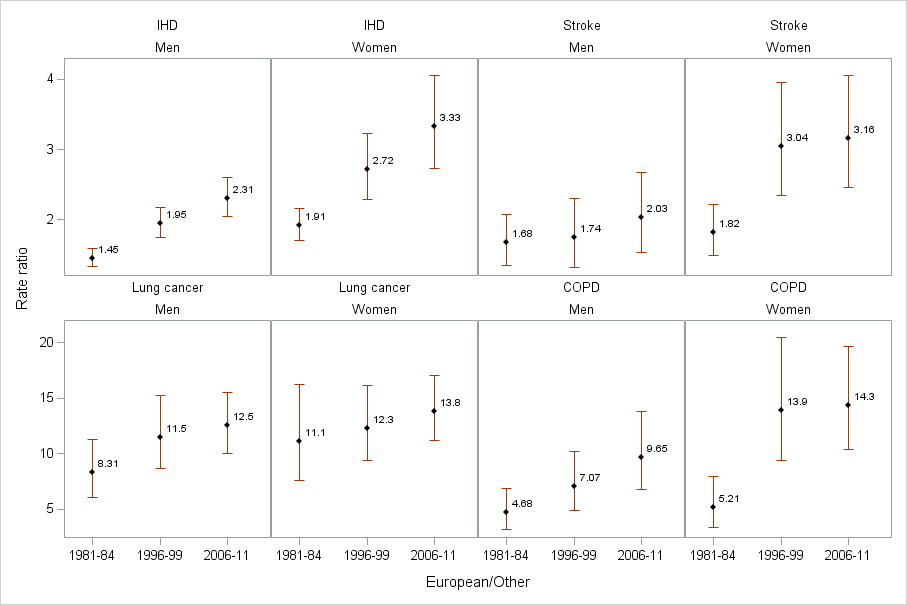


Table S4: Rates and rate differences (per 100,000) in all-cause mortality for current and never smokers estimated from the Poisson regression model adjusted for socioeconomic position and household smoking. Rates are presented for the ‘average’ man or woman of European/Other ethnicity who is 60-64 years old, with school qualifications, lives in an area of average deprivation, has a mean level of income, is employed, owns a house, has access to one car, and is not exposed to household smoking; from the New Zealand Census Mortality Study. We then carry out the same process for Māori and European/Other models that were run separately in this population, allowing for interaction by ethnicity.

|  | **Poisson estimates** |  |  | **Men** |  |  | **Women** |  |
| --- | --- | --- | --- | --- | --- | --- | --- | --- |
|  |  | **Cohort** | 1981-84 | 1996-99 | 2006-11 | 1981-84 | 1996-99 | 2006-11 |
| **Total** | Rate | Current-smoker | 1639 | 1283 | 1041 | 779 | 705 | 630 |
|  |  | Never-smoker | 1107 | 724 | 582 | 515 | 391 | 332 |
|  | Rate difference |  | 533 | 559 | 459 | 264 | 314 | 298 |
|  | Rate ratio |  | 1.48 | 1.77 | 1.79 | 1.51 | 1.80 | 1.90 |
| **Māori** | Rate | Current-smoker | 1267 | 1760 | 1507 | 951 | 1126 | 962 |
|  |  | Never-smoker | 1305 | 1308 | 1176 | 864 | 962 | 695 |
|  | Rate difference |  | -38 | 452 | 332 | 87 | 164 | 267 |
|  | Rate ratio |  | 0.97 | 1.35 | 1.28 | 1.10 | 1.17 | 1.38 |
| **European/Other** | Rate | Current-smoker | 1736 | 1354 | 1113 | 817 | 741 | 678 |
|  |  | Never-smoker | 1118 | 708 | 555 | 513 | 367 | 316 |
|  | Rate difference |  | 619 | 646 | 559 | 304 | 374 | 361 |
|  | Rate ratio |  | 1.55 | 1.91 | 2.01 | 1.59 | 2.02 | 2.14 |

Figure S7: Poisson estimated mortality rate differences (RDs) and rate ratios (RRs) in current and never smokers run separately for Māori and European/Other populations. RDs and RRs are presented for the ‘average’ man and woman who is 60-64 years old, with school qualifications, lives in an area of average deprivation, has a mean level of income, is employed, owns a house, has access to one car, and is not exposed to household smoking; in the New Zealand Census Mortality Study.


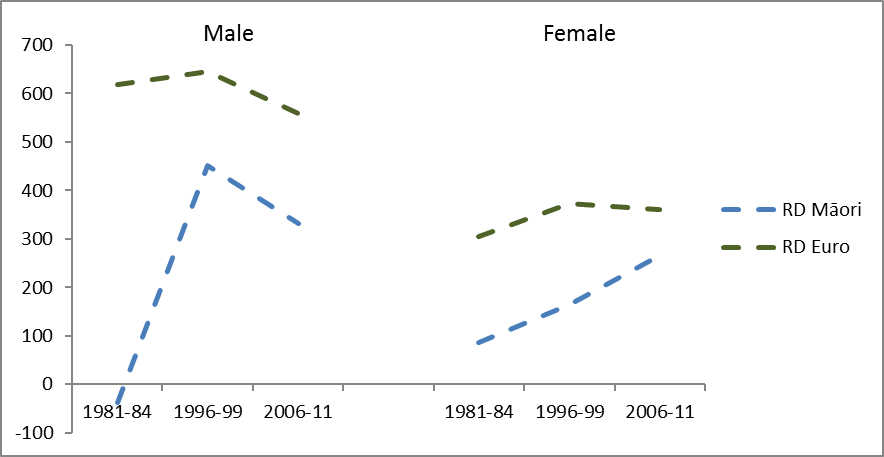

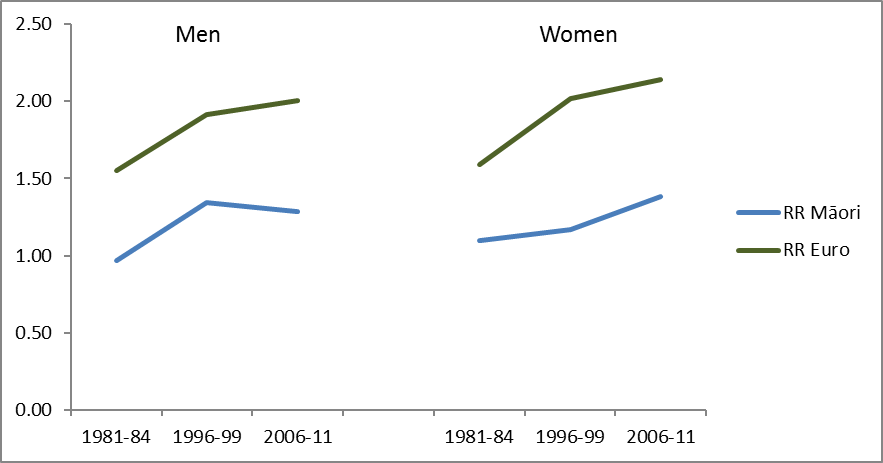


**Table S5: Full results for the Poisson regression analysis estimating the rate ratio (RR) association comparing mortality in current-smokers with never-smokers over time in 25-74 year olds (yo), New Zealand Census Mortality Study (95%CIs in brackets). The final Poisson model (model 3) adjusted (adj) for age, ethnicity, income, education, deprivation index, labour force status, car access, tenure and household smoking (HHS).**

| RRs |  | Men |  |  | Women |  |  | Combined |  |  |
| --- | --- | --- | --- | --- | --- | --- | --- | --- | --- | --- |
|  |  | 1981-84 | 1996-99 | 2006-11 | 1981-84 | 1996-99 | 2006-11 | 1981-84 | 1996-99 | 2006-11 |
| Age & ethnicity adj (1) | RR (no restrictions) | 1.64 (1.57-1.71) | 2.01 (1.92-2.10) | 2.18 (2.10-2.26) | 1.58 (1.50-1.65) | 1.98 (1.88-2.09) | 2.25 (2.15-2.34) | 1.63 (1.58-1.68) | 2.00 (1.94-2.07) | 2.20 (2.14-2.26) |
|  | RR* | 1.63 (1.55-1.72) | 2.12 (2.02-2.23) | 2.24 (2.14-2.33) | 1.60 (1.51-1.69) | 2.08 (1.96-2.20) | 2.30 (2.19-2.42) | 1.64 (1.58-1.70) | 2.12 (2.04-2.20) | 2.26 (2.19-2.33) |
|  | % change in excess RR | - | 75% | 95% | - | 80% | 117% | - | 73% | 95% |
| Age, ethnicity & SEP adj (2) | RR* | 1.51 (1.44-1.59) | 1.82 (1.73-1.91) | 1.85 (1.77-1.93) | 1.54 (1.45-1.63) | 1.87 (1.77-1.99) | 1.97 (1.87-2.07) | 1.54 (1.48-1.60) | 1.85 (1.78-1.93) | 1.89 (1.83-1.96) |
|  | % change in excess RR | - | 61% | 67% | - | 61% | 80% | - | 57% | 65% |
| Age, ethnicity, SEP & HHS adj (3) | RR* | 1.48 (1.40-1.56) | 1.77 (1.68-1.87) | 1.79 (1.71-1.87) | 1.51 (1.42-1.61) | 1.80 (1.69-1.92) | 1.90 (1.80-2.00) | 1.51 (1.45-1.57) | 1.79 (1.72-1.87) | 1.83 (1.77-1.89) |
|  | % change in excess RR | - | 60% | 65% | - | 57% | 76% | - | 55% | 63% |
| By ethnicity | Māori (1) | 1.08 (0.90-1.29) | 1.63 (1.43-1.86) | 1.63 (1.46-1.82) | 1.20 (0.97-1.49) | 1.39 (1.19-1.63) | 1.77 (1.56-2.01) | 1.13 (0.98-1.29) | 1.51 (1.37-1.68) | 1.67 (1.54-1.82) |
|  | Māori (2) | 1.00 (0.84-1.21) | 1.39 (1.22-1.60) | 1.32 (1.18-1.48) | 1.15 (0.92-1.43) | 1.22 (1.04-1.43) | 1.45 (1.27-1.65) | 1.05 (0.92-1.21) | 1.31 (1.18-1.45) | 1.36 (1.25-1.48) |
|  | Māori (3) | 0.97 (0.81-1.17) | 1.35 (1.17-1.54) | 1.28 (1.14-1.44) | 1.10 (0.88-1.37) | 1.17 (1.00-1.37) | 1.38 (1.21-1.58) | 1.02 (0.88-1.17) | 1.25 (1.13-1.39) | 1.31 (1.20-1.43) |
|  | European/Other (1) | 1.71 (1.62-1.80) | 2.29 (2.17-2.42) | 2.53 (2.41-2.65) | 1.67 (1.57-1.77) | 2.33 (2.18-2.48) | 2.61 (2.47-2.76) | 1.71 (1.64-1.77) | 2.32 (2.23-2.42) | 2.57 (2.48-2.66) |
|  | European/Other (2) | 1.58 (1.50-1.67) | 1.96 (1.86-2.08) | 2.07 (1.97-2.17) | 1.61 (1.51-1.71) | 2.10 (1.96-2.24) | 2.21 (2.09-2.34) | 1.60 (1.54-1.67) | 2.03 (1.95-2.12) | 2.13 (2.05-2.21) |
|  | European/Other (3) | 1.55 (1.47-1.64) | 1.91 (1.80-2.03) | 2.01 (1.91-2.11) | 1.59 (1.50-1.70) | 2.02 (1.89-2.16) | 2.14 (2.02-2.27) | 1.58 (1.52-1.64) | 1.97 (1.89-2.06) | 2.07 (1.99-2.15) |
|  |  |  |  |  |  |  |  |  |  |  |
| By mortality type (3) | IHD | 1.42 (1.30-1.55) | 1.75 (1.58-1.93) | 2.03 (1.83-2.25) | 1.86 (1.65-2.08) | 2.38 (2.03-2.78) | 2.90 (2.44-3.44) | 1.54 (1.44-1.65) | 1.91 (1.76-2.08) | 2.19 (2.01-2.39) |
|  | Stroke | 1.59 (1.30-1.95) | 1.53 (1.20-1.96) | 1.76 (1.39-2.23) | 1.73 (1.43-2.09) | 2.41 (1.89-3.07) | 2.75 (2.20-3.44) | 1.71 (1.49-1.97) | 1.97 (1.65-2.34) | 2.24 (1.91-2.63) |
|  | Lung cancer | 7.84 (5.87-10.5) | 9.83 (7.72-12.5) | 9.71 (8.11-11.6) | 8.73 (6.16-12.4) | 10.0 (7.88-12.8) | 11.0 (9.16-13.2) | 8.50 (6.81-10.6) | 10.1 (8.51-12.0) | 10.4 (9.14-11.8) |
|  | COPD | 4.30 (3.00-6.16) | 6.70 (4.76-9.43) | 6.71 (4.98-9.02) | 4.74 (3.19-7.03) | 10.1 (7.15-14.2) | 11.0 (8.28-14.5) | 4.60 (3.53-5.98) | 8.76 (6.88-11.2) | 8.99 (7.33-11.0) |
| Maori (3) | IHD | 1.00 (0.71-1.41) | 1.11 (0.84-1.47) | 1.35 (1.05-1.74) | 1.25 (0.77-2.05) | 1.36 (0.93-2.00) | 2.08 (1.40-3.10) | 1.05 (0.80-1.39) | 1.17 (0.94-1.47) | 1.49 (1.20-1.84) |
|  | Stroke | 0.84 (0.38-1.88) | 0.85 (0.42-1.72) | 1.13 (0.61-2.12) | 1.42 (0.73-2.79) | 0.77 (0.39-1.52) | 2.04 (1.11-3.75) | 1.14 (0.68-1.91) | 0.80 (0.49-1.31) | 1.50 (0.98-2.31) |
|  | Lung cancer | 4.82 (1.91-12.2) | 5.48 (3.31-9.08) | 8.26 (4.89-13.9) | 2.67 (1.12-6.37) | 5.15 (2.83-9.37) | 5.56 (3.45-8.96) | 3.51 (1.87-6.58) | 5.32 (3.62-7.82) | 6.84 (4.81-9.73) |
|  | COPD | 3.01 (1.01-8.99) | 7.03 (2.32-21.3) | 2.63 (1.33-5.19) | 2.51 (0.90-7.02) | 2.92 (1.36-6.26) | 3.28 (1.77-6.09) | 2.80 (1.33-5.88) | 4.13 (2.28-7.50) | 2.97 (1.88-4.69) |
| European/ Other (3) | IHD | 1.45 (1.33-1.59) | 1.95 (1.75-2.18) | 2.31 (2.05-2.60) | 1.91 (1.70-2.16) | 2.72 (2.29-3.23) | 3.33 (2.73-4.05) | 1.58 (1.47-1.69) | 2.14 (1.95-2.35) | 2.50 (2.25-2.77) |
|  | Stroke | 1.68 (1.35-2.08) | 1.74 (1.32-2.30) | 2.03 (1.54-2.68) | 1.82 (1.49-2.22) | 3.04 (2.34-3.96) | 3.16 (2.47-4.06) | 1.78 (1.54-2.06) | 2.36 (1.95-2.86) | 2.58 (2.15-3.11) |
|  | Lung cancer | 8.31 (6.11-11.3) | 11.5 (8.66-15.2) | 12.5 (10.1-15.5) | 11.1 (7.56-16.2) | 12.3 (9.39-16.1) | 13.8 (11.2-17.0) | 9.54 (7.50-12.1) | 12.0 (9.91-14.6) | 13.4 (11.5-15.3) |
|  | COPD | 4.68 (3.18-6.88) | 7.07 (4.89-10.2) | 9.65 (6.75-13.8) | 5.21 (3.40-7.99) | 13.9 (9.40-20.5) | 14.3 (10.4-19.7) | 5.00 (3.76-6.64) | 10.6 (8.08-13.9) | 12.3 (9.68-15.6) |
|  |  |  |  |  |  |  |  |  |  |  |
| By age (1) | 25-44 yo (1) | 1.27 (1.09-1.47) | 1.83 (1.59-2.10) | 1.39 (1.23-1.58) | 1.09 (0.90-1.33) | 1.29 (1.08-1.54) | 1.47 (1.27-1.70) | 1.20 (1.06-1.34) | 1.61 (1.44-1.79) | 1.41 (1.28-1.55) |
|  | 45-64 yo (1) | 1.73 (1.60-1.87) | 2.17 (2.01-2.35) | 2.31 (2.17-2.45) | 1.65 (1.51-1.80) | 2.00 (1.83-2.19) | 2.16 (2.01-2.32) | 1.71 (1.62-1.82) | 2.11 (2.00-2.24) | 2.24 (2.13-2.34) |
|  | 65-74 yo (1) | 1.66 (1.54-1.79) | 2.18 (2.03-2.35) | 2.57 (2.41-2.75) | 1.68 (1.54-1.82) | 2.38 (2.19-2.60) | 2.81 (2.61-3.03) | 1.69 (1.60-1.79) | 2.30 (2.18-2.44) | 2.68 (2.55-2.81) |
| By age (2) | 25-44 yo (2) | 1.17 (1.00-1.36) | 1.52 (1.32-1.76) | 1.12 (0.98-1.27) | 1.04 (0.85-1.27) | 1.09 (0.91-1.32) | 1.14 (0.98-1.34) | 1.11 (0.98-1.25) | 1.35 (1.21-1.51) | 1.12 (1.02-1.24) |
|  | 45-64 yo (2) | 1.60 (1.48-1.74) | 1.83 (1.69-1.99) | 1.85 (1.73-1.97) | 1.57 (1.44-1.72) | 1.81 (1.65-1.98) | 1.81 (1.68-1.95) | 1.61 (1.52-1.71) | 1.84 (1.73-1.95) | 1.83 (1.75-1.92) |
|  | 65-74 yo (2) | 1.54 (1.43-1.66) | 1.90 (1.77-2.05) | 2.19 (2.05-2.34) | 1.63 (1.50-1.77) | 2.18 (2.00-2.37) | 2.50 (2.32-2.70) | 1.60 (1.51-1.69) | 2.05 (1.94-2.17) | 2.32 (2.20-2.44) |
| By age (3) | 25-44 yo (3) | 1.12 (0.96-1.31) | 1.52 (1.31-1.76) | 1.12 (0.97-1.29) | 1.01 (0.83-1.24) | 1.10 (0.91-1.34) | 1.12 (0.95-1.31) | 1.07 (0.94-1.21) | 1.35 (1.20-1.51) | 1.11 (1.00-1.24) |
|  | 45-64 yo (3) | 1.56 (1.43-1.69) | 1.79 (1.65-1.94) | 1.80 (1.69-1.93) | 1.56 (1.43-1.71) | 1.72 (1.57-1.89) | 1.74 (1.62-1.88) | 1.58 (1.49-1.68) | 1.78 (1.68-1.89) | 1.78 (1.69-1.87) |
|  | 65-74 yo (3) | 1.53 (1.41-1.65) | 1.84 (1.71-1.99) | 2.08 (1.94-2.23) | 1.59 (1.46-1.74) | 2.08 (1.91-2.27) | 2.41 (2.23-2.61) | 1.58 (1.49-1.67) | 1.98 (1.87-2.09) | 2.22 (2.11-2.33) |

# Appendix D: Sensitivity Tests

We tested for selection bias by estimating smoking RRs from the Poisson age and ethnicity-adjusted model for an unrestricted population (usual resident, private dwelling, not absent on census night) and compared this to the study sample who additionally had no missing SEP and HHS variables. The smoking-mortality RR in the restricted analysis were slightly greater than model estimates for the complete population with no missing SEP and HHS covariates (excess RRs ranged from 2% less to 10% greater, Table S6). That is there may be a modest selection bias such that RRs in the main paper are slightly overestimated.

A negative control was used to test for the impact of residual confounding. We examined standardised mortality rates for causes of death not strongly causally associated with smoking (i.e. suicide and unintentional injury). The age and ethnicity adjusted smoking-mortality RRs for unintentional injury were attenuated with adjustment for SEP, resulting in no significant association with smoking in men at any time point, but injury was associated with smoking in women for all time points, with the greatest RR in 1996-99 (Supplementary Table S7).

It is possible that reverse causality biased results towards the null (Table S8). Excluding the first 12 months of follow-up aims to avoid some of the bias where people quit smoking because they are unwell from cancer or heart disease in their final year of their life. This test reduced the fully-adjusted Poisson RR estimates by 8-25% in men and women, and the effect was greatest in men in the 1981-84 cohort. Thus, while our analyses may be biased to overestimate the smoking-mortality RR, this bias appears greatest in the earliest cohort, meaning we have actually understated the increase in smoking RRs over time.

The follow-up in the 2006-11 cohort was longer than the earlier cohorts. We tested whether this impacted the results, by limiting this cohort to the same 36 month follow-up as the other cohorts. This test resulted in smoking-RRs that were 5% less in men and 2% less in women (Table S9) and did not markedly change the study results.

**Table S6: Sensitivity test for selection bias comparing unrestricted results with study results restricted to individuals with complete data on all variables. Models are adjusted for age and ethnicity only. (95% confidence intervals in brackets)**

|  | **Men** |  |  | **Women** |  |  |
| --- | --- | --- | --- | --- | --- | --- |
|  | **1981-84** | **1996-99** | **2006-11** | **1981-84** | **1996-99** | **2006-11** |
| RR no restrictions | 1.64  (1.57-1.71) | 2.01  (1.92-2.10) | 2.18  (2.10-2.26) | 1.58  (1.50-1.65) | 1.98  (1.88-2.09) | 2.25  (2.15-2.34) |
| RR restricted to complete data | 1.63  (1.55-1.72) | 2.12  (2.02-2.23) | 2.24  (2.14-2.33) | 1.60  (1.51-1.69) | 2.08  (1.96-2.20) | 2.30  (2.19-2.42) |
| % change in excess RR | 2% | -10% | -5% | -3% | -10% | -4% |

**Table S7: Sensitivity test presenting the rate ratios comparing current-smokers and never-smokers for unintentional injury and suicide mortality, 25-74 year olds, New Zealand Census Mortality Study. Model 1 adjusts for age and ethnicity. Model 2 adjusts for age, ethnicity and socioeconomic position. Model 3 is the full Poisson model adjusted for age, ethnicity, socioeconomic position and household smoking.**

|  | | **Men** | | | **Women** | | |
| --- | --- | --- | --- | --- | --- | --- | --- |
|  | | **1981-84** | **1996-99** | **2006-11** | **1981-84** | **1996-99** | **2006-11** |
|  | **Unintentional injury** | |  |  |  |  |  |
|  | Model 1 | 1.14 (0.84-1.54) | 1.40 (1.02-1.90) | 1.36 (1.10-1.69) | 2.00 (1.17-3.41) | 4.19 (2.18-8.04) | 2.16 (1.58-2.96) |
|  | Model 2 | 1.05 (0.77-1.42) | 1.25 (0.90-1.72) | 1.10 (0.88-1.38) | 1.91 (1.11-3.28) | 3.78 (1.94-7.38) | 1.82 (1.32-2.52) |
|  | Model 3 | 1.09 (0.80-1.50) | 1.19 (0.85-1.66) | 1.10 (0.87-1.39) | 1.94 (1.11-3.36) | 4.19 (2.12-8.27) | 1.86 (1.33-2.61) |
|  | **Suicide** |  |  |  |  |  |  |
|  | Model 1 | 1.38 (1.02-1.86) | 2.83 (2.25-3.56) | 2.75 (2.29-3.30) | 1.79 (1.17-2.74) | 2.05 (1.35-3.13) | 3.10 (2.29-4.19) |
|  | Model 2 | 1.31 (0.96-1.78) | 2.50 (1.98-3.18) | 2.45 (2.02-2.97) | 1.78 (1.15-2.74) | 1.91 (1.24-2.96) | 2.76 (2.01-3.79) |
|  | Model 3 | 1.33 (0.97-1.83) | 2.55 (1.99-3.26) | 2.62 (2.15-3.20) | 1.75 (1.12-2.74) | 2.19 (1.40-3.42) | 2.83 (2.03-3.93) |

**Table S8: Sensitivity test for reverse causality by excluding the 12 months of follow-up for deaths that occurred within 12 months of when smoking status was collected in the census, 25-74 year olds, New Zealand Census Mortality Study**

|  | **Men** |  |  | **Women** |  |  |
| --- | --- | --- | --- | --- | --- | --- |
|  | **1981-84** | **1996-99** | **2006-11** | **1981-84** | **1996-99** | **2006-11** |
| Fully adjusted Poisson model | 1.48 (1.40-1.56) | 1.77 (1.68-1.87) | 1.79 (1.71-1.87) | 1.51 (1.42-1.61) | 1.80 (1.69-1.92) | 1.90 (1.80-2.00) |
| Fully adjusted Poisson model: excluding first 12m follow-up | 1.60 (1.51-1.71) | 1.91 (1.79-2.03) | 1.85 (1.76-1.95) | 1.56 (1.45-1.67) | 1.86 (1.73-2.00) | 1.99 (1.88-2.11) |
| Proportion increase in excess smoking-mortality rate ratio | 25% | 18% | 8% | 10% | 8% | 10% |

**Table S9: Sensitivity test of greater mismeasurement bias in the 2006-11 cohort due to longer follow-up, in fully adjusted Poisson model, 25-74 year olds, New Zealand Census Mortality Study**

|  | **2006-11 cohort** | | |
| --- | --- | --- | --- |
|  | **Men** | **Women** | **Combined** |
| Full follow-up 60 months | 1.79 (1.71-1.87) | 1.90 (1.80-2.00) | 1.83 (1.77-1.89) |
| Limited follow-up to 36 months | 1.75 (1.65-1.85) | 1.88 (1.75-2.01) | 1.79 (1.71-1.87) |
| Proportion change in excess smoking-mortality rate ratio | -5% | -2% | -5% |
